# *Fusobacterium nucleatum* Lipopolysaccharides O‑Antigen Defines a Novel Siglec‑7 Binding Epitope

**DOI:** 10.1021/jacsau.5c00810

**Published:** 2025-09-25

**Authors:** Cristina Di Carluccio, Ferran Nieto-Fabregat, Linda Cerofolini, Celeste Abreu, Luis Padilla-Cortés, Giulia Roxana Gheorghita, Alessandro Antonio Masi, Lorena Buono, Manasik Gumah Adam Ali, Dimitra Lamprinaki, Antonio Molinaro, Nathalie Juge, Giovanni Smaldone, Ondřej Vaněk, Marco Fragai, Roberta Marchetti, Alba Silipo

**Affiliations:** 1 Department of Chemical Sciences, University of Naples Federico II, Via Cinthia 4, Naples 80126, Italy; 2 CEINGE-Biotecnologie Avanzate Franco Salvatore, Via Gaetano Salvatore 486, Napoli 80145, Italy; 3 Magnetic Resonance Centre (CERM), CIRMMP and Department of Chemistry “Ugo Schiff”, 9300 University of Florence, Via Luigi Sacconi 6, Sesto Fiorentino 50019, Italy; 4 Department of Biochemistry, Faculty of Science, Charles University, Hlavova 2030/8, Prague 12800, Czech Republic; 5 Giotto Biotech s.r.l., Sesto Fiorentino 50019, Italy; 6 IRCCS SYNLAB SDN, Via G. Ferraris 144, Naples 80146, Italy; 7 The Food, Microbes and Health Institute Strategic Programme, Norwich Research Park, Quadram Institute Bioscience, Norwich, NR4 7UQ U.K.

**Keywords:** Siglec-7, *Fusobacterium nucleatum*, NMR, molecular dynamics

## Abstract

*Fusobacterium nucleatum* (*Fn*) is a Gram-negative bacterium predominantly found in the human oral cavity, occasionally linked to systemic diseases, including colorectal cancer. Bacterial lipopolysaccharides (LPSs) represent one of the possible virulence factors contributing to and promoting disease progression. *Fn* LPS is recognized by Siglec-7, a sialic acid-binding inhibitory receptor expressed on immune cells and promising novel target for cancer immunotherapy. Through a combined approach of structural biology, biophysics, NMR, and computational methods, we explored the molecular basis of the interaction between Siglec-7 and the LPS from*F. nucleatum* *ssp polymorphum* 10953, whose O-antigen contains peculiar sugars such as the neuraminic acid and the AAT (FucpNAc4N). We discovered a novel Siglec-7 binding epitope within the LPS O-antigen repeating unit, defined by its internal sialic acid and AAT residues. We propose a wing-like movement of the O-antigen, where Siglec-7 BC and CC’ loops alternately engage the O-antigen edges within the binding site, with the BC loop forming more stable interactions. We uncover a novel *Fn*10953 immune evasion mechanism and highlight Siglec-7 and LPS as novel therapeutic targets for *Fn*-associated CRC, providing new avenues for intervention.

## Graphical abstract

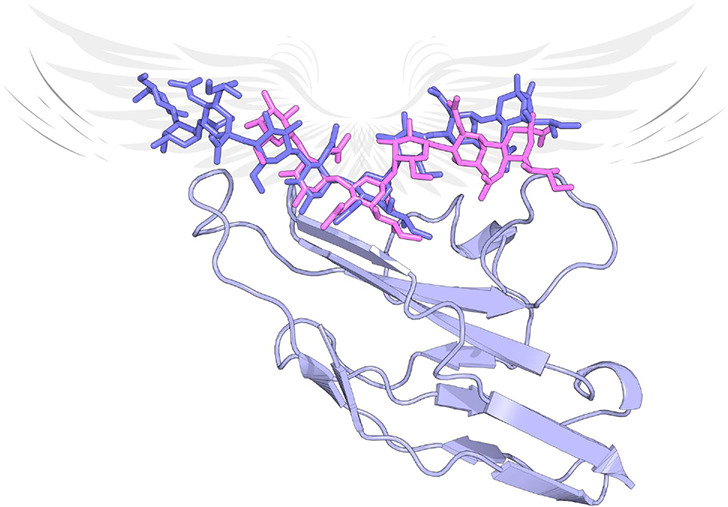

## Introduction

*Fusobacterium nucleatum* ^1,2^ is a Gram-negative obligate anaerobe, among the most abundant species residing in the human oral cavity and rarely found in the lower gastrointestinal (GI) tract of healthy individuals.^1^ *Fn* has been identified as one of the pathobionts that outgrow during dysbiosis preceding periodontal disease.^2^ *Fusobacterium* can disseminate systemically in different body sites, such as the genitourinary tracts, and overcome placental and blood–brain barriers. A large number of studies suggest that *Fn* is closely related to the developments of systemic diseases, including rheumatoid arthritis, Alzheimer’s disease, adverse pregnancy outcomes, and GI disorders such as IBD.^3^ In addition, *Fn* has gained attention as emerging cancer-associated bacteria, overabundant in various types, including colorectal (CRC), pancreatic, esophageal, and breast cancers,^4,5^ and associated with shorter patients survival. In particular, CRC is associated with gut microbiota dysbiosis characterized by a reduction in protective or beneficial bacteria such as *Clostridium* and *Faecalibacterium* and an increase in cancer-associated polyketide synthase*Escherichia coli*, enterotoxigenic*Bacteroides fragilis* and *Fn*.^2,6,7^ *Fn* promotes CRC progression via several mechanisms, including inhibition of host antitumor immunity, innate immune cell modulation, activation of cell proliferation, promotion of cellular invasion, induction of chronic inflammation and immune evasion, and mobilization of immune cells in the tumor microenvironment.^7^ ^,^ ^8^

A virulence factor in *Fn* is the lipopolysaccharide (LPS), a microbe-associated molecular pattern located on the outer membrane of Gram-negative bacteria. LPS is an amphiphilic molecule with a general structural architecture composed of (i) a glycolipid moiety named lipid A and (ii) a heteropolysaccharide portion containing the core oligosaccharide and the O-antigen polysaccharide. Structural studies on the LPS of *Fn* have identified a wide array of carbohydrate components, including sialic acids and/or unusual sugars like fusaminic acid,^3^ highlighting the complexity of its glycome. These sugars contribute to the unique immunogenic properties of the bacterium, playing critical roles in its pathogenicity and immune evasion.

The sialic acid-binding immunoglobulin-type lectins (Siglecs) comprise a family of 15 cell surface proteins, mostly inhibitory receptors, which can be divided into two different classes according to their sequence and evolutionary similarity: the main subset of CD33-related Siglecs and the conserved Siglecs’ subgroup (Scheme S1). Siglecs mostly recognize sialic acid (Sia) containing glycans through a key conserved arginine residue in the N-terminal domain that establishes a salt bridge with the carboxylate group of Sia (Scheme S1). Therefore, engagement of sialylated glycoprotein and glycolipids on all mammalian cells by Siglecs induces tolerance to self-antigens and prevents unwanted autoimmune responses. However, hypersialylation, a frequent trait of tumor cells, increases sialoglycan–Siglec interactions to evade immune surveillance in the favorable immunosuppressive tumor microenvironments, making Siglecs a novel emerging immune checkpoint for cancer therapy.^9^

Interestingly, several pathogens, including bacteria like *E. coli* K1, *Neisseria meningitidis*, *N. gonorrheae*, group B *Streptococcus* (GBS), and *Campylobacter jejuni*, and viruses, such as HIV-1, SARS-CoV-2, Ebola virus, have evolved cell-surface Sia mimicry as a mechanism for engaging Siglecs, thus attenuating host immune responses and promoting dissemination. This mimicry is achieved in bacteria through envelope components (*e.g*., sialylated capsules and/or LPS) or, in some cases, modified flagellin.^10,11^

Siglec-7 (CD328), a member of the CD33-related Siglecs subgroup, is primarily expressed on the surface of innate lymphoid human NK cells and is also found on dendritic cells.^12^ It exhibits a binding preference for α2,8-linked disialylated ligands and internally branched α2,6-sialyl residues. Siglec-7 consists of an extracellular region, containing an N-terminal V-set domain representing the carbohydrate binding region (Figure S1), two C2-type Ig-like domains, and an immunoreceptor tyrosine-based inhibitory (ITIM) motif in the cytosolic region (Scheme S1).^13^ Siglec-7 *trans* interaction with cognate ligands drives the phosphorylation of its cytoplasmic ITIM domain that triggers the inhibition of NK cell pathways. Tumor cells and pathogens can exploit this mechanism to evade immune recognition and facilitate their migration through the circulatory system. For example, GBS surface protein β binds Siglec-7 on NK cells, inhibiting pyroptosis and suppressing NK cell sentinel activity.^14^ The selective recognition of sialylated glycans exposed on *C. jejuni* lipooligosaccharides by Siglec-7 has also been reported and potentially related to the clinical outcome and the development of secondary complications such as Guillain-Barré syndrome.^15^ *Pseudomonas aeruginosa* sialylation contributes to bacterial pathogenicity and interaction with the host via reduction of complement deposition and with Siglec-dependent recognition. These interactions demonstrate that pathogens, including *Fusobacterium nucleatum*, can exploit Siglec-mediated immune modulation as a shared strategy across microbial and viral systems.^16^ By leveraging sialic acid recognition, these pathogens suppress immune activation and evade host defenses, promoting successful colonization and persistence in diverse pathological contexts such as cancer and infectious diseases.^11^

We previously showed that *F. nucleatum* *ssp. animalis* ATCC51191, the most predominant species in CRC, interacts with Siglec-7 expressed on innate immune cells, and that this interaction also occurred with purified bacterial outer membrane vesicles and LPS.^17^ This interaction induced a pro-inflammatory profile in human monocyte-derived dendritic cells and a tumor associated profile in human monocyte-derived macrophages, likely contributing to tumor progression, and unveiled LPS O-antigen as a potential ligand for Siglec-7. Specifically, we focused on *F. nucleatum* ssp. *polymorphum* 10953 (*Fn*10953), a strain frequently isolated from colorectal cancer tissues, whose LPS O-antigen possesses a unique repeating unit including peculiar sugars as the neuraminic acid residue and the AAT (FucpNAc4N) moiety (Scheme 1)^18^; the precise molecular mechanisms by which this O-antigen is recognized by human Siglec-7 have yet to be elucidated. To unravel this essential host–pathogen interaction, we employed a powerful combination of complementary approaches, including biochemical and biophysical techniques, comprehensive NMR spectroscopy from both protein and ligand perspectives, and advanced computational modeling. This integrated strategy allowed us to fully characterize the binding of *Fn*10953 LPS to Siglec-7, defining a previously unrecognized and novel molecular recognition mode.

**Scheme 1:**
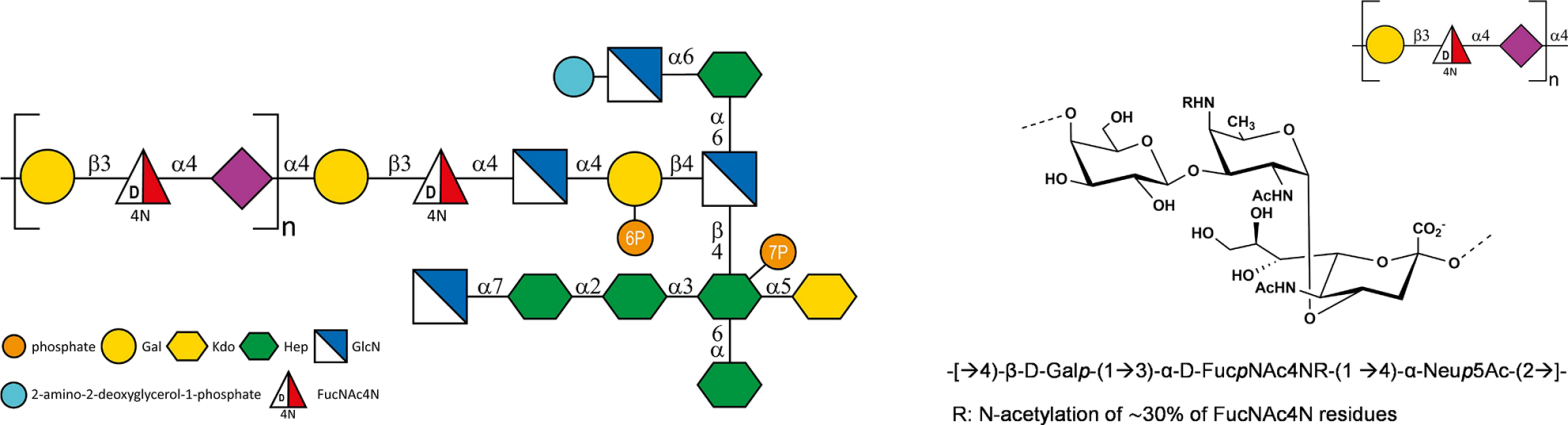
SNFG Schematic Representation of *Fn*10953 LPS Structure^39^ ^a^

a (A) OPS containing the O-antigen repeating unit and the core oligosaccharide.^40^ (B) O-Antigen trisaccharide repeating unit as derived by the mild acid hydrolysis of *Fn*10953 O-polysaccharide chain: →4)-β-D-Galp-(1→3)-α-D-FucpNAc4NR-(1→4)-α-Neup5Ac-(2→; R = H (up to 75%), R = Ac (25-30%)

## Results

The full extracellular domain (FED) of Siglec-7 was expressed in HEK293 GnTI- (human embryonic kidney) cells.^19^ The LPS from *Fn*10953 was extracted and purified following established methodology, confirming the composition of the O-antigen repeating unit (Scheme 1). Notably, the acetylation of the Fuc*p*NAc4N (2-acetamido-4-amino-2,4,6-trideoxy-D-galactose, AAT) unit at N4 was around 25–30%, as previously reported.^18^ Furthermore, a series of oligomers containing an increasing number of O-antigen (OPS) repeating units were generated through mild acid hydrolysis (Scheme 1) exploiting the lability of the sialic acid glycosidic linkage. These oligomers were employed to elucidate the molecular basis of *Fn*10953 O-antigen recognition and binding by Siglec-7.

### *Fn*10953 LPS Binds Siglec-7 and Differentially Activates Blood Cells

To assess the recognition of *Fn*10953 LPS by Siglec-7, we first evaluated its binding to cells overexpressing Siglec-7. To this end, multiple hematopoietic cell lines were subjected to flow cytometric analysis using a PE-conjugated anti-Siglec-7 antibody (see methods for details). Among these cell lines, RPMI8226, a well-established model of multiple myeloma, demonstrated the highest surface expression of Siglec-7 (Figure S3). To assess the interaction of *Fn*10953 LPS and its isolated O-antigen with Siglec-7, RPMI8226 cells were incubated with increasing concentrations of these ligands. Subsequently, binding to Siglec-7 was evaluated by flow cytometry using a PE-conjugated anti-Siglec-7 antibody. As shown in Figure 1A, both *Fn*10953 LPS and its O-antigen bound Siglec-7-expressing RPMI8226 cells (IC50 of approximately 2 μg/mL for LPS and 18 μg/mL for OPS), while no binding was detected with *Salmonella* LPS, used as a negative control.

**Figure 1:**
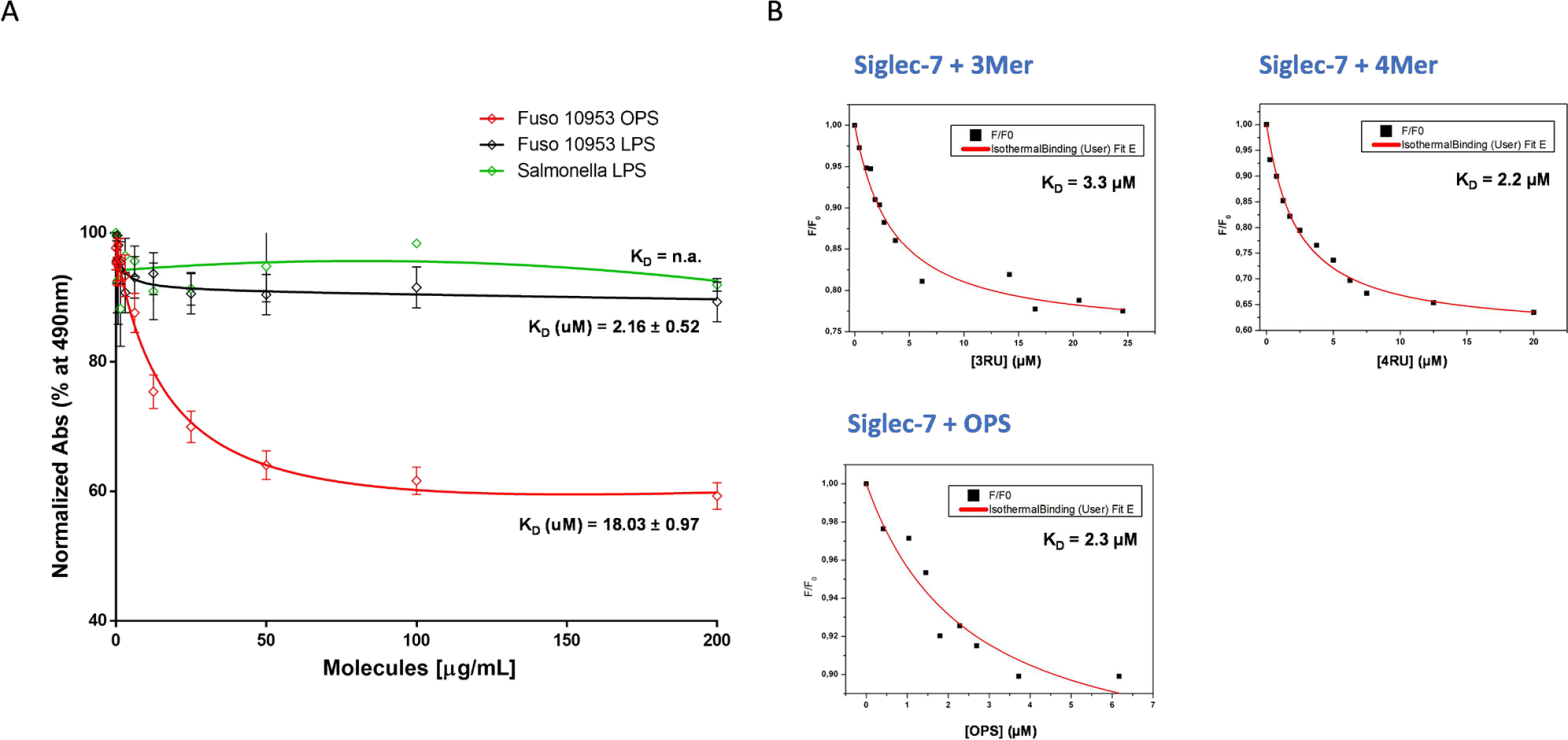
Recognition of *Fn*10953 LPS by Siglec-7 via ELISA and Fluorescence Analysis: (A) ELISA binding assay of *Salmonella* LPS (green line), *Fn*10953 LPS (black line), and *Fn*10953 full polysaccharide (OPS) (red line) with RPMI8226 cell line. The *K*_D_ (μM) and the corresponding *R* ^2^ values are reported in the figure. The lower/poorer quality of the LPS binding data could be due to the steric cluster generated by this molecule, which could cover the binding sites generating a worse signal than OPS. (B) Fluorescence analysis of Siglec-7 with different oligomers from *Fn*10953. The quenching fluorescence titration of Siglec-7 with *Fn*10953 3-Mer, *Fn*10953 4-Mer, and *Fn*10953 full polysaccharide (OPS) were fitted using a nonlinear regression with the one site-specific binding model for the determination of the association constants *K*_b_ (μM^–1^) and the corresponding *K*_D_ (μM).

Next, steady-state fluorescence analysis was conducted to determine the binding affinities of Siglec-7 for the *Fn*10953 O-antigen. We observed a concentration-dependent reduction in fluorescence intensity of Siglec-7 upon glycan addition. The binding constants (*K*_b_) were determined by nonlinear regression analysis (Figure 1B).^20^ No change in fluorescence intensity was observed when using the trisaccharide containing a single repeating unit (1-Mer), suggesting that it was insufficient for Siglec-7 recognition (Figure S4A). In contrast, titration with longer oligomers, containing up to four repeating units (4-Mer), resulted in quenching of Siglec-7 tryptophan residues, indicative of complex formation. These interactions exhibited comparable low-micromolar *K*_D_ similar to that observed for the entire LPS O-antigen (Figure 1B).

The effect of *Fn*10953 LPS and its O-antigen on the levels of Siglec-7 surface expression in blood cells was evaluated using flow cytometry. As expected, the results demonstrated that NK cells exhibited the highest expression of Siglec-7 (Figure 2A and Figure S5). The ability of *Fn*10953 LPS to activate individual blood components was first investigated using peripheral blood mononuclear cells (PBMCs) from 5 healthy donors by measuring HLA-DR expression levels. HLA-DR, a key class II MHC molecule involved in antigen presentation to CD4+, serves as a marker of immune activation. T-lymphocytes, B-lymphocytes, and NK cells were identified, and HLA-DR expression levels on the gated cells were simultaneously assessed (as described in the gating strategy in Figure S6). As shown in Figure 2B, a significant increase in HLA-DR positive cells, compared to untreated cells, was observed for *Fn*10953 O-antigen on NK cells; the other combinations of treatments were not significant (Table S1). T-lymphocytes, B-lymphocytes, and NK cells were then purified and subsequently activated with both *Fn*10953 LPS and O-antigen. No significant changes were observed in the degree of activation of T-lymphocytes (Figure 2C) and B-lymphocytes (Figure 2D). Significant changes in NK cell activation were observed following treatment with *Fn*10953 O-antigen compared to untreated NK cells or those treated with Fn10953 LPS or *Salmonella* LPS, consistent with the findings observed in PBMCs (Figure 2E). These data, although preliminary, suggest the involvement of Siglec-7 in the activation process of NK cells in the presence of *Fn*10953 O-antigen.

**Figure 2:**
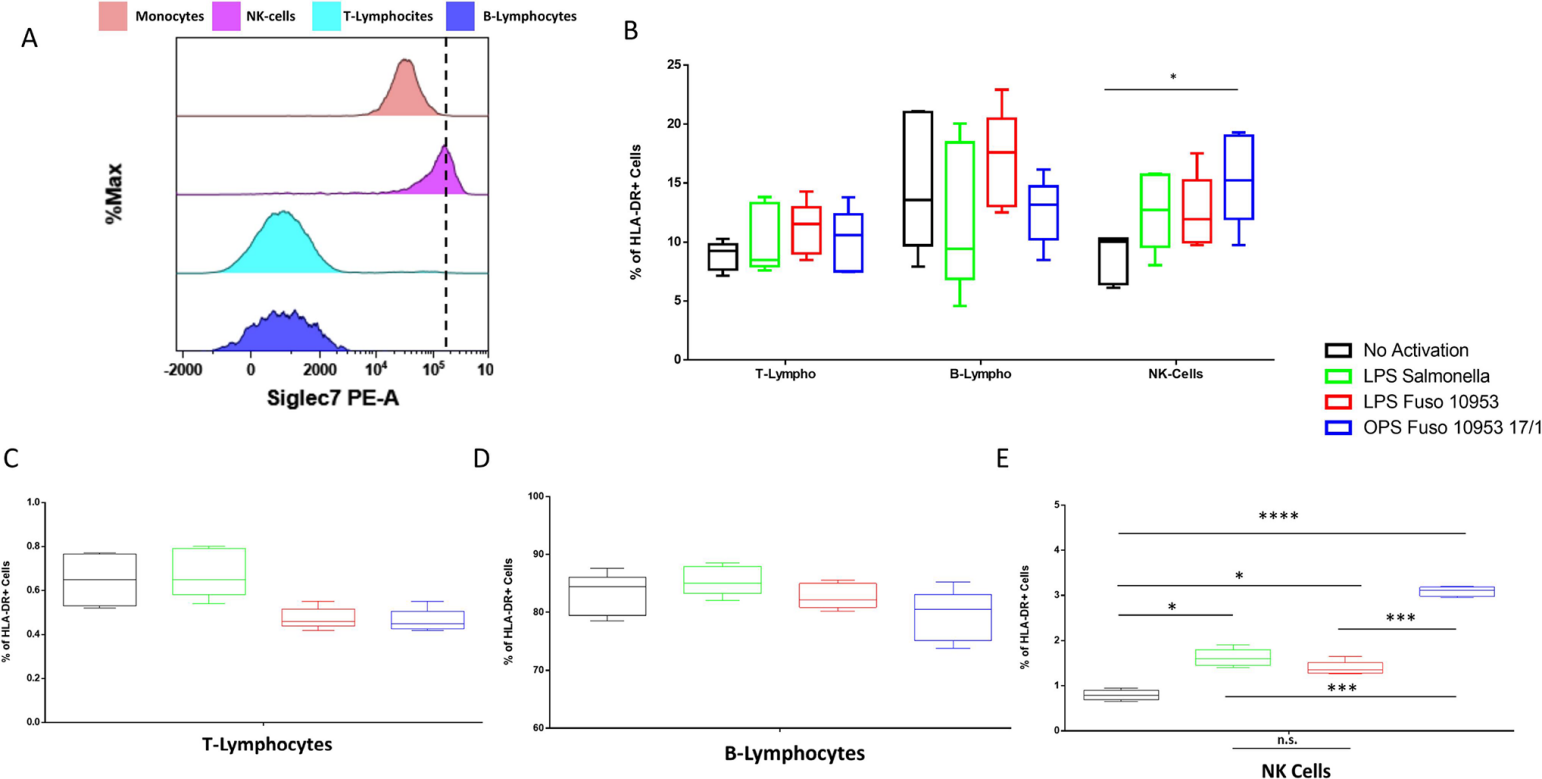
Siglec-7 protein expression on blood cells. (A) Cytofluorimetric analyses of Siglec-7 protein levels in monocytes (pink), NK cells (magenta), T-lymphocytes (light blue), and B-lymphocytes (blue). Percentage of HLA-DR+ cells in PBMC derived from 5 healthy donors (B) and in purified B-lymphocytes (C), T-lymphocytes (D), and NK cells (E) from the same subjects. Black bars represent untreated cells. Green bars represent *Salmonella* LPS-treated cells. Red bars represent Fn10953 LPS-treated cells. Blue bars represent *Fn*10953 O-antigen treated cells. *p-value <0.05, ***p-value <0.001, ****p-value <0.0001; n.s., not significant. Paired *t* test.

### Molecular Details of Siglec-7 and *Fn*10953 O-Antigen Oligomers

#### Ligand-Based NMR Binding Studies

The molecular recognition features of *Fn*10953 LPS by Siglec-7 were elucidated through a combined approach of NMR spectroscopy and MD (molecular dynamics) simulations. Ligand-based NMR techniques, including saturation transfer difference (STD), transferred NOESY (tr-NOESY), and relaxation experiments, were employed to investigate the molecular interactions between Siglec-7 and *Fn*10953 O-antigen oligomers. These experiments elucidated the ligand binding epitopes and their conformational behavior upon binding.^21^ In accordance with fluorescent binding assays (Figure S4A), no STD NMR effects were observed between Siglec-7 and 1-Mer (data not shown), indicating the absence of binding. This finding confirms that a single repeating unit of the *Fn*10953 O-antigen is insufficient for recognition and binding by Siglec-7. Conversely, NMR binding experiments conducted on the core-OPS fraction revealed selective binding of Siglec-7 to the O-antigen moiety. Notably, no STD NMR effects were observed from protons belonging to the core region (Figure S4B).

To determine the minimal epitope required for O-antigen binding to Siglec-7, NMR experiments were performed with *Fn*10953 OPS oligomers. A first indication of complex formation was obtained by comparing the CPMG experiments of the 3-Mer alone and in the mixture with the protein. Indeed, a decrease of signals in the bound state and differences in the transverse relaxation time *T*_2_ values (Table S2) were detected upon binding.

STD NMR analysis carried out on the isolated 2-Mer (Figure 3A) revealed that the recognition primarily occurred through the internal sialic acid (**N’**) and the AAT (FucpNAc4N) (**A’**) residues (Figure 2A). Protons at positions 4, 5, and 7 of **N’** exhibited clear STD NMR enhancements, different from the reducing sialic acid unit **N**. Furthermore, a slight STD NMR enhancement observed for **N’3**_**eq**_ provided further evidence for Siglec-7 preference for the internal sialic acid residue; additionally, protons at positions 6 and 9 exhibited significant magnetization transfer, further corroborating the crucial role of the **N’** unit in the interaction with Siglec-7. Moreover, while STD signal intensities of the acetyl groups of **N**, **A** and **A’** residues were quite low, a robust STD response was detected for the acetyl group of **N’**. Regarding the recognition of the AAT moiety, a strong STD signal was observed for the anomeric proton of **A’** (the internal AAT), while residue **A** exhibited no binding signals. Protons at positions 2 and 6 of **A’** displayed moderate STD effects, whereas the remaining protons of **A’** showed STD signals below 20%. These findings collectively demonstrate the involvement of the internal AAT **A’** in binding to Siglec-7. In contrast, galactose units showed no STD NMR signals, demonstrating that they are not involved in the binding process and away from the protein binding pocket (Figure 3A). Moreover, no STD contribution was detected from the proton at position 4 of AAT when acetylated, likely suggesting that the low degree of OPS acetylation does not influence or participate in the general binding to Siglec-7. Together, these data showed that the *Fn*10953 2-Mer preferentially accommodated the internal sialic acid and not acetylated AAT residues into the binding site of Siglec-7.

**Figure 3:**
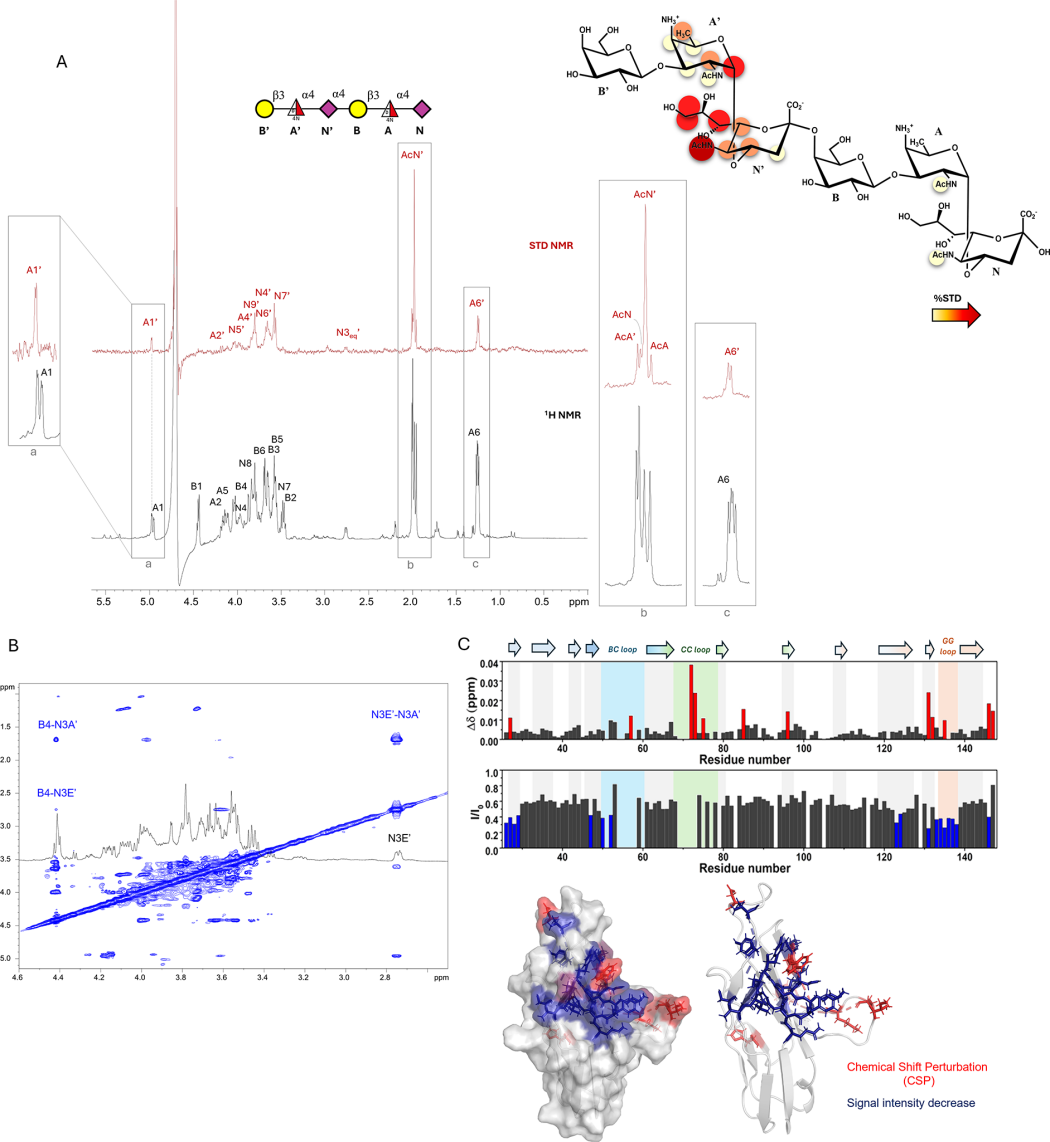
NMR studies of the interaction between *Fn*10953 OPS and Siglec-7. (A) Schematic structure of the 2-Mer ligand depicted following the Symbol Nomenclature for Glycans (SNFG^39^). STD NMR spectrum composed of ^1^H NMR reference spectrum (bottom) and 1D STD NMR spectrum (top), of the Siglec-7:2-Mer mixture at a 1:50 ratio. 2D representation illustrating the interacting epitope map resulted from STD NMR data, showcasing the interactions between 2-Mer and Siglec-7. (B) tr-NOESY spectrum of the Siglec-7:2-Mer mixture at a 1:50 ratio. The key NOE contacts shown in the spectrum were indicative of extended bioactive conformation of 2-Mer. (C) Diagrams of the CSP (top) and decrease in signal intensity (bottom) of the amino acids of 200 μM Siglec-7 in the presence of 4 mM OPS from *Fn*-10953. The CSP effects were evaluated with the formula $\Delta \delta =\frac{1}{2}\sqrt{\Delta {\delta_{H}}^{2}+{\left(\Delta \delta_{N}/5\right)}^{2}}$ . The residues experiencing the largest CSP (S27, D57, I72, S73, K75, W85, H96, K131, W132, K135, L146, and T147) have been highlighted in red; the residues experiencing the largest decrease in signal intensity (Y26, S27, L28, T29, S47, Y50, V52, F123, R124, K131, N133, Y134, K135, Y136, D137, Q138, and L146) have been highlighted in blue. On the right, the 3D surface of Siglec-7 with the amino acids experiencing the largest CSP colored in red and those with the largest decrease in signal intensity in blue.

To further investigate the binding mode, tr-NOESY NMR and computational studies were combined to generate a 3D model of Siglec-7 and 2-Mer complex. Comparing NOESY (Figure S7A) and tr-NOESY spectra (Figure 3B), no substantial differences were observed in the NOE contacts or in the interproton distances, indicating that the hexasaccharide containing two repeating units underwent no substantial conformational changes upon binding. Notably, the presence of key NOEs, as the contact of **B4–N’3** (axial and equatorial) and **B1–A2** (Figure 3B and Figure S7B), as well as the absence of the NOE between **N’3** with **A5′** and **A6’** identified a ligand conformational preference toward the *t* conformation (φ = 180°, Figure S7B). To depict a 3D complex, the uncommon AAT sugar residue was parametrized using the AMBER18 package^22^; then, the 2-Mer was built according to the energetic minima calculated through the adiabatic maps by molecular mechanics calculation (see details in Supporting Information, Figure S8A) and subjected to MD simulation (Figure S8B). The ligand in the free state exhibits conformational flexibility, primarily ascribable to φ (C1–C2–O-C4’) torsion angle around Neu5Ac-α-(2,4)-Gal glycosidic linkage (Figure S8B), that could populate two different values, corresponding to 180° (conformer *t*) and −60° (conformer -*g*), which lead to an extended and folded shape, respectively (Figure S8C). In contrast, the ψ (C1–O–C4’–H4’) torsion angle remains relatively stable, maintaining a value around 0°. In detail, the MD simulations (Figure S8B,C) corroborated the NOE-based findings. Cluster analysis of the free ligand trajectories revealed that it predominantly resides in the extended *t* conformation (φ = 180°) for approximately 70% of the simulation time. This computational evidence further supports the notion that the 2-Mer preferentially adopts an extended conformation in its free state. Similarly and according to tr-NOESY experiments, the 2-Mer adopted an extended conformation upon binding (Figure S8D).

An almost comparable binding epitope (Figure S9) and bioactive conformation were detected for 3-Mer. This suggests that Siglec-7 accommodates and recognizes the 3-Mer in a similar manner.

#### Protein-Based NMR Binding Studies

To further elucidate the protein–ligand interaction, protein-based NMR experiments were conducted to identify the glycan binding region on the Siglec-7 surface (Figure 3C). To characterize the complex, a series of NMR titrations were performed and increasing concentrations of 3-Mer were added to a solution of [U-15N]-labeled Siglec-7. The analysis of chemical shift perturbation (CSP) and/or decrease in signal intensity of cross-peaks in the 2D ^1^H–^15^N HSQC spectra enabled the mapping of the Siglec-7 binding pocket. The addition of the ligand mainly induced a decrease in signal intensity, and the residues experiencing the largest decrease in signal intensity were the following: Tyr26, Ser27, Leu28, Thr29, Ser47, F123, R124, Lys131, and Asn133, as well as Tyr50 and Val52 of BC loop, and the amino acids Tyr134, Lys135, Tyr136, Asp137, and Gln138 of the GG’ loop. Otherwise, Ile72, Ser73, and Lys75 of the CC loop were affected by CSP, together with Ser27, Trp85, His96, Lys131, Trp132, Leu146, Thr147, and Asp57 of the BC loop as well as Lys135 of the GG’ loop. These results indicate that the key Arg124 and the neighboring amino acids (from Lys131 to Gln138) form the main binding site of Siglec-7, with residues from BC and CC’ loops partially involved in the binding with the O-antigen oligomers.

#### Siglec-7–Fn10953 O-Antigen 3D Complex

The extended, bioactive conformation of 2-Mer was modeled in the V-set Siglec-7 binding site (PDB 2HRL^23^) and once optimized with Maestro Schrödinger software, the complex was subjected to minimizations and MD simulations (Figure 4).^24^ The results demonstrated the stability of the complex (Figure 4D) and confirmed that 2-Mer preferentially accommodated in the bound state with an extended conformation, in full agreement with the tr-NOESY studies. MD results indicated that the 3D complex of Siglec-7 with *Fn*10953 2-Mer exhibited significant contacts primarily mediated by residues **N’** and **A’**. These findings closely aligned with the NMR results (Figure 3). The carboxylate moiety of **N’** played a crucial role in the interaction, forming a stable salt bridge with the guanidinium group of Arg124. This salt bridge emerged as the most prominent interaction throughout the MD simulation (Figure 4), effectively anchoring and orienting the glycan within the binding site. Further interactions were engaged by **N’** with Asn133 and Lys131 residues side chains, both located in the GG’ loop, through the hydroxyl group at position 8 of the glycerol chain and the NAc moiety, respectively. Additionally, the hydroxyl group at position 9 interacted with both Asn133 and Lys135 residues. On the other hand, Lys131 simultaneously stabilized **A’** residue by interacting with its acetyl group. Notably, the interaction between Siglec-7 and the AAT residue (**A’**) further involved Asn129, which formed polar contacts with the positively charged amino group at position 4. Furthermore, Asn70 and Trp74 of the CC’ loop established transient hydrogen bonds interactions with the NAc moiety of the reducing **N** residue. Overall, these computational studies combined with STD and tr-NOESY NMR results, indicated that the 2-Mer adopted an extended conformation, with residues **N’** and **A’** playing crucial roles in the interaction with Siglec-7, engaged in polar, charged, and hydrophobic interactions.

**Figure 4:**
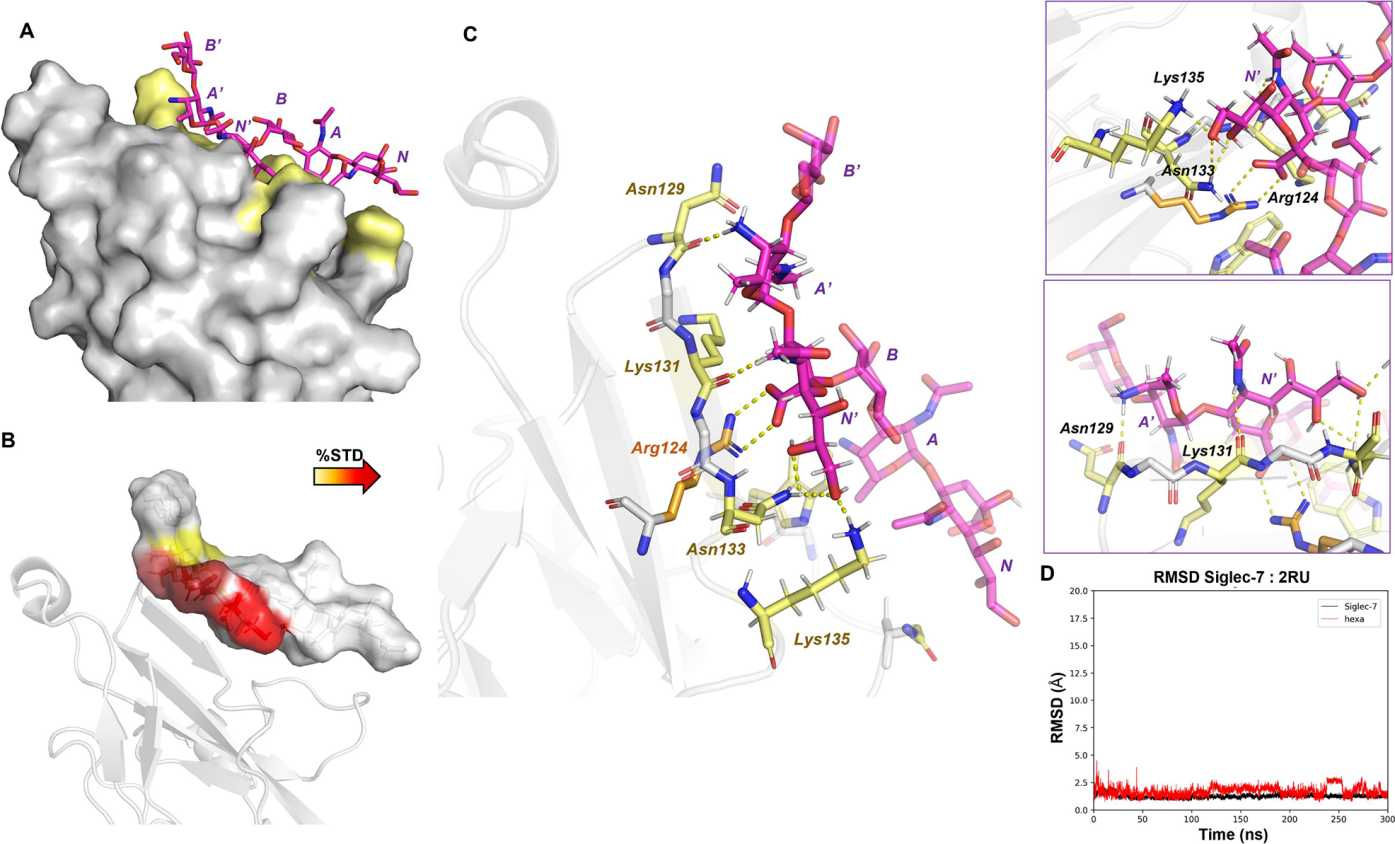
Analysis of MD simulation of the Siglec-7:2-Mer complex: (A) 3D model of the Siglec-7:2-Mer complex, showing the protein surface with the interacting amino acids highlighted in yellow. (B) 3D depiction of the interaction, with the ligand surface colored based on STD NMR and computational findings. (C) 3D representation of a dominant pose from the most prevalent MD simulation cluster. The primary amino acids involved in binding are highlighted in yellow, with the key Arg124 in orange. The ligand is depicted in magenta, indicating a preference for an extended conformation in the MD simulations. Dashed yellow lines represent observed hydrogen bond interactions. A detailed view illustrates the interactions between Siglec-7 and the sialic acid (N’) and AAT (A’) units. (D) Protein (black) and ligand (red) RMSD of the Siglec-7:2Mer system. The ligand RMSD was calculated in reference to the protein.

To construct a 3D model of the Siglec-7/3-Mer complex, the nonasaccharide was modeled into the receptor binding pocket. This positioning was guided by the experimental data, which indicated that the reducing sialic acid unit (**Sia-1**) did not interact with the protein. Consequently, the **Sia-1** residue was oriented away from the protein surface during the model building process. Conversely, both **Sia-2** and **Sia-3** sialic acid units had the potential to interact with Arg124 within the binding site. On the one hand, when **Sia-3** engages with Arg124, the ligand extends predominantly toward the CC’ loop; conversely, when **Sia-2** interacts with Arg124, the saccharide chain extends toward both the CC’ and BC loops of the protein. This interaction mode maintained potential contacts with the CC’ loop while additionally allowing for possible interactions with the BC loop. Furthermore, MD simulations were conducted using both the *g* and *t* conformations of the ligand, exploring various orientations within the binding site. The MD results demonstrated that, as expected, the ligand preferentially adopted the *t* conformation around the Sia-Gal linkage. Other torsion angles adopted values consistent with the *exo-syn* anomeric effect. Furthermore, the most stable interactions throughout the MD simulations involved the internal trisaccharide epitope (Figure 5A–E and Figure S10). Indeed, the carboxylate group of **Sia-2** served as a key anchor in the interaction with Arg124, forming the most stable interaction observed throughout the MD simulation. In addition to Arg124, Asn133, and Lys131 stabilized the hydroxyl group at position 8 and the NAc group of **Sia-2**, respectively. Furthermore, the Lys131 carbonyl oxygen of the peptide bone acted as a hydrogen bond acceptor in the interaction with the acetyl group of **AAT-2**. Similarly, the carbonyl oxygen of the Asn129 peptide backbone served as a hydrogen bond acceptor in its interaction with the positively charged amino group of **AAT-2** mirroring interactions observed in the Siglec-7–2-Mer complex. Moreover, in the Siglec-7/3-Mer complex, the ammonium ion at N4 of **AAT-2** also interacted with Asp53, part of the BC loop that acted as a hydrogen bond acceptor.

**Figure 5:**
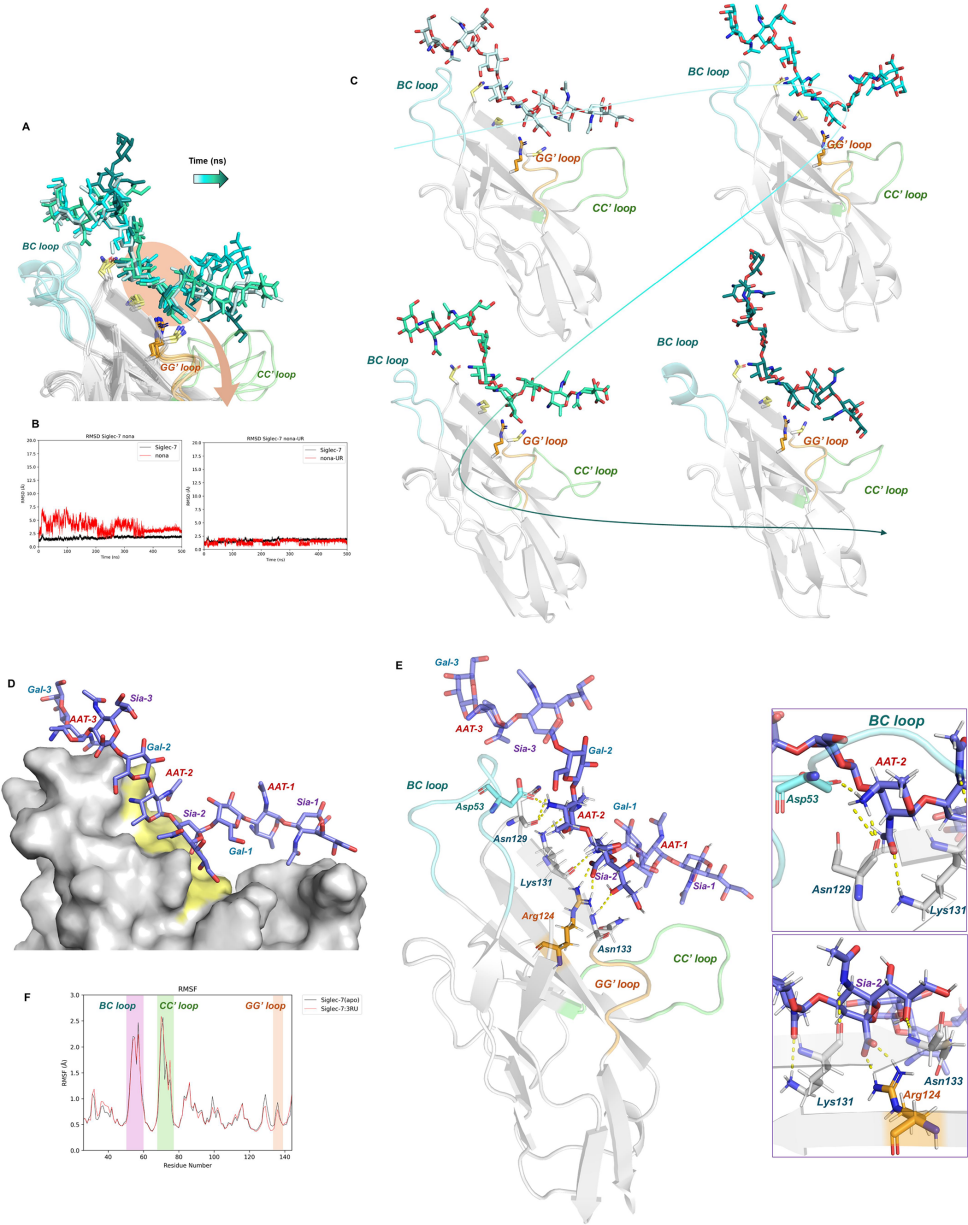
Dynamics and stability of the Siglec-7/3-Mer complex across MD simulations. (A) 3D visualization of the Siglec-7/3-Mer complex through the overlay of various representative poses (from light to dark blue) to depict the mobility of the complex during MD, with the 3-Mer ligand shaded in varying tones of blue over time. The BC, CC’ and GG’ loops are highlighted in cyan, green, and orange, respectively. Amino acids involved in 3-Mer recognition are marked in yellow, with the key Arg124 in orange. (B) Left: RMSD analysis of the Siglec-7/3-Mer system; right: RMSD of the inner trisaccharide (Gal2-AAT2-Sia2), illustrating the internal trisaccharide’s stability during MD simulations, in contrast to the 3-Mer’s variability due to the movements of terminal and reducing trisaccharides. (C) Temporal representation showcasing simultaneous movements of both terminal and reducing trisaccharides along with the BC and CC’ loops, highlighting the anchored internal trisaccharide amidst ‘wing-like’ movements. (D) 3D model of the Siglec-7/3-Mer complex showing the protein surface with interacting amino acids in yellow. (E) 3D view of a representative pose from the most common MD simulation cluster, with the amino acids involved in the binding colored in gray, Arg124 in orange, and Asp53 from the BC loop in cyan. BC, CC’, and GG’ loops are again depicted in cyan, green, and orange, respectively, with dashed yellow lines indicating hydrogen bond interactions. A closer inspection reveals the interactions between Siglec-7 and the sialic acid (Sia-2, bottom) and AAT (AAT-2, top). (F) RMSF results showing the protein flexibility in the free (black) and bound (red) states. A slight decrease flexibility is observed only for the GG’ loop.

Finally, analysis of root mean square fluctuations (RMSF) during the MD simulation indicated that the BC and CC’ loops exhibited a high degree of flexibility even in the bound state. In contrast, the GG’ loop demonstrated a decrease in flexibility upon ligand binding (Figure 5F). This could be explained by the close proximity of GG’ loop residues to the binding pocket cavity, where the critical interactions with the internal trisaccharide epitope occur. Thus, while the internal repeating unit remained stable during the MD, establishing polar interactions with Arg124, Asn129, Lys131, and Asn133, the BC and CC’ loop acted as wings allowing the formation of polar and hydrophobic interactions with the terminal and reducing trisaccharide, respectively (Figure 5).

## Discussion

*F. nucleatum*, an oral bacterium strongly implicated in periodontitis, is also highly prevalent in various gastrointestinal diseases, including colorectal cancer (CRC). Emerging evidence strongly indicates that *Fn* plays a significant role in tumorigenesis, facilitating tumor growth, promoting metastasis, and modulating host immune responses to favor tumor progression. Recent findings suggests that *F. nucleatum* localization within tumors is primarily mediated by glycan-lectin interactions.^25^ Specifically, the surface-exposed Fap2 lectin of Fn recognizes the Gal-GalNAc epitope (the Thomsen–Friedenreich antigen), which is abundantly overexpressed on the surface of colorectal and breast cancer cells.^4^ In addition, we previously reported that *Fn*10953 interacts with Siglec-7 on immune cells through the LPS. In this study, we employed a multidisciplinary approach, encompassing fluorescence spectroscopy, NMR spectroscopy, molecular modeling, and biological assays, to comprehensively investigate the molecular mechanisms underlying the interaction between *Fn*10953 LPS with Siglec-7. We showed that *Fn*10953 LPS, along with its isolated O-antigen, specifically binds to Siglec-7 on RPMI8226 cells, a multiple myeloma model. *Salmonella* LPS, as a negative control, did not show binding, confirming specificity. Among blood cells, Siglec-7 is highly expressed on NK cells, aligning with its known role in modulating NK cell functions. Exposure of PBMCs from healthy donors to *Fn*10953 LPS O-antigen significantly upregulated HLA-DR expression on NK cells. In contrast, T-lymphocytes and B-lymphocytes did not exhibit significant activation. Purified NK cells exhibited significant activation responses to both *Fn*10953 LPS and its O-antigen, in contrast to T-lymphocytes and B-lymphocytes, which showed minimal activation. The higher level of NK cell activation induced by *Fn*10953 LPS O-antigen suggests a potent immunostimulatory role mediated by specific Siglec-7 engagement.

The ability of *Fusobacterium nucleatum* to engage Siglec-7 exemplifies a shared strategy among pathogens to evade immune surveillance through sialic acid-dependent interactions. This mirrors the findings in reproductive infections and viral pathogenesis, where Siglecs are co-opted to modulate immune responses and promote pathogen survival.^11^ The ‘double-edged sword’ nature of Siglec’ interactions is particularly evident with *Fusobacterium nucleatum*, as its immune modulation via Siglec-7 engagement parallels strategies observed in viruses such as HIV and SARS-CoV-2.^16^ This conserved mechanism of immune evasion highlights the potential of *Fn* to profoundly impact the tumor microenvironment by modulating immune responses.

At the molecular level, our data demonstrated that the core region of the LPS did not contribute to Siglec-7 binding. Moreover, equilibrium affinity constants for the entire O-antigen and for oligosaccharides containing varying numbers of repeating units were comparable, all ranging within the micromolar range (Figures 3C and 6). Furthermore, the single trisaccharide repeating unit (1-Mer) was found to be insufficient for interaction with Siglec-7. This observation suggests that a longer glycan chain is necessary to induce recognition by Siglec-7. We identified the internal sialic acid **N’** and the AAT residue **A’** as key contributors to the binding process, while the galactose unit did not significantly interact with Siglec-7. Notably, the 2-Mer, which preferentially adopted an extended conformation in the free state (Figures 3B and 4 and Figure S7), maintained this conformation upon binding to Siglec-7. Extensive MD simulations confirmed the ligand’s preference for the *t* conformation around the Sia glycosidic linkage (φ = 180°). The internal Sia residue **N’** was accommodated in the binding pocket establishing the crucial salt bridge between its carboxylate group and the key Arg124 residue, as well as hydrogen bonds with its NHAc and the glycerol chain and Lys131, Asn133, and Lys135 residues. Additionally, significant interactions were observed between the positively charged amino group at N4 of AAT **A’** and the carbonyl group of the Asn129 backbone. The above lead to the overall stabilization of the protein GG’ and CC’ loops upon binding.

**Figure 6:**
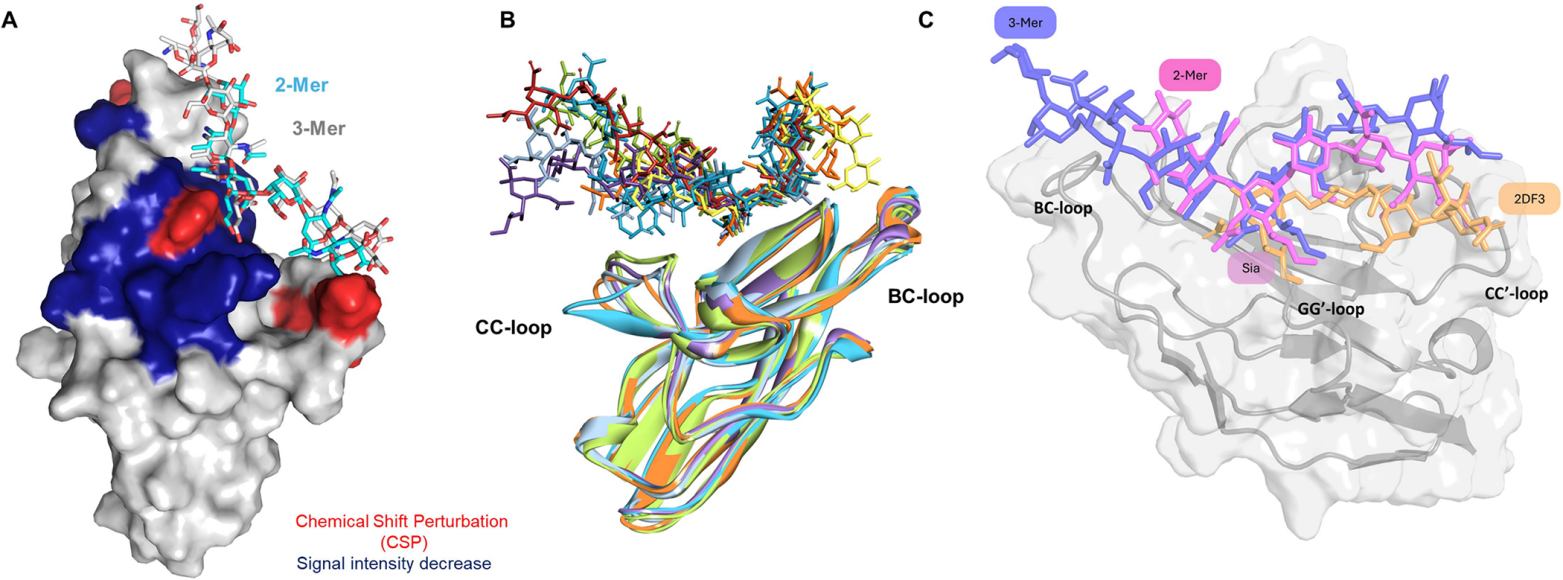
3D views of Siglec-7 in complex with 2-Mer and 3-Mer. (A) Superimposition of the complexes of Siglec-7 in interaction with 2-Mer (cyan) and 3-Mer (gray); the amino acids of Siglec-7 are colored according to protein-based NMR titration with 3-Mer. (B) Superimposition of the different poses of Siglec-7 in interaction with 3-Mer obtained by MD simulation. (C) Comparison of ligand binding conformations within the BC-loop and CC'-loop regions. The DSLc4 ligand (orange) is shown alongside the 2-Mer (pink) and 3-Mer (purple) ligands. Differences in their positioning on the protein surface highlight the distinct interactions each ligand forms within the binding site, especially in relation to the BC-loop and CC’-loop.

The ligand containing three repeating units (3-Mer) demonstrated a preferential interaction with Siglec-7 primarily through **Sia-2**, favoring its engagement with Arg124 within the Siglec-7 binding site compared to the other two Sias residues. Both **Sia-2** and **AAT-2** emerged as the key determinants of ligand recognition, and induced a chemical shift perturbation and an intensity decrease in the neighboring amino acids, as evidenced by protein-based NMR experiments (Figures 3B and 6) and corroborated by their stability in the RMSD analysis (Figure 5B). Thus, signals of Arg124 and the contiguous/adjacent amino acids, comprising Lys131, Asn133, and Lys135, were affected by the intensity decrease and were also identified as the main residues in contact with **Sia-2** and **AAT-2** of the 3-Mer oligosaccharide. Notably, Lys135, together with Tyr134, Tyr136, Asp137, and Glu138 belong to the GG’ loop, which is slightly stabilized in the bound state, as monitored by the RMSF (Figure 5F). Notably, protein-based NMR experiments performed on the full *Fn*10953 O-antigen (OPS) identified the key interaction sites of Siglec-7 that corroborated findings with the 3-Mer. Computational analyses suggested a dynamic binding interface, with the BC and CC’ loops exhibiting a “wing-like” movement, potentially interacting with different regions of the O-antigen. However, the BC loop appeared to form more stable interactions with the ligand compared to the CC’ loop, which exhibited more transient contacts. Indeed, from protein-based NMR results, in the BC loop, Tyr50 and Val52 were affected by an intensity decrease and Asp57 was subjected to a CSP; instead, the CC’ loop was only affected by the CSP effects detected for Ile72 and Lys76, thus involved in a faster exchange with the ligand.

Therefore, the specific conformational features of the *Fn*10953 LPS O-antigen likely play a crucial role in Siglec-7 binding. Through presentation of an appropriate epitope within the Sia-AAT unit, the O-antigen promotes optimal accommodation within the Siglec-7 binding site. This binding mode was compared to the accommodation of the disialylated ganglioside DSLc4 in the binding site (crystal structure 2DF3), where the terminal sialic acid linked to GlcNAc interacts extensively with the GG’ loop, whereas the terminal Sia linked to Gal interacts with the CC’ loop. Conversely, *Fn*10953 LPS O-antigen accommodation inside the binding site is driven by internal sialic acid units that preferentially interact with the Siglec-7 BC loop (Figure 6C and Figure S11).

We recently demonstrated that *Neisseria meningitidis* serogroup Y capsular polysaccharide targets the inhibitory receptor Siglec-7 through a binding mode crucially dependent on the specific arrangement of its sialic acids and the 3D structure, thereby revealing a key mechanism where bacterial glycans exploit host inhibitory pathways to facilitate immune evasion.^26^

Here, our comprehensive investigation further provides crucial insights for understanding disease mechanisms and host–pathogen interactions. We clearly demonstrate a novel binding mode for the interaction between *Fn*10953 LPS and Siglec-7. This unique recognition mechanism is fundamentally driven by the intricate three-dimensional arrangement of the Fn10953 O-antigen. Specifically, this mode highlights the pivotal role of internal sialic acid residues, integral component of the O-antigen repeating unit and responsible for mediating stable complexes through key electrostatic and polar interactions. This discovery thus delineates a new paradigm for glycan-mediated bacterial exploitation of host inhibitory receptors. Finally, we provide critical insights into how *Fusobacterium nucleatum* LPS could modulate immunity via Siglec-7, contributing to immune suppression, evasion strategies, and altered immune cell recognition. Beyond this understanding, this study implications are profound, strongly suggesting the therapeutic potential of targeting Siglec-7 for both CRC and other related conditions.

## Methods

### Healthy Subjects and Cell Line

Healthy subjects enrolled in this study were enrolled according to the protocol approved by IRCCS Pascale, Institutional Ethical Review Board (CE: Protocollo n. 4/21, 2021). RPMI 8226 cell line were obtained by IRCCS SYNLAB SDN Biobank (10.5334/ojb.26) and cultured in RPMI supplemented with 10% fetal bovine serum and 1% Glutamine. For the interaction study, 3 × 10^4^ cells/well were seeded in a 96 well plate and incubated at 37 °C and 5% CO_2_. After o/n incubation different concentration of LPS from *Salmonella*, *Fusobacterium nucleatum* and OPS from *Fusobacterium nucleatum* were added (from 0.39 μg/mL to 200 μg/mL) for 1 h at 37 °C. After two PBS washes, cells were labeled with anti-Siglec-7 antibody PE-conjugated (#12–5759–42, Invitrogen). After appropriate wash, a minimum of 10,000 events were recorded at Cytoflex cytofluorimeter (Beckman coulter). For the interaction analysis, the normalized (respect to the untreated sample) mean fluorescence intensities of the anti-Siglec7 antibody were reported with respect to the concentrations of LPS and OPS. Experiments were repeated three times with similar results. The k_D_s were calculated using GraphPad 6 software (One-site Total equation). For the blood cells separation, T-lymphocytes, B-lymphocytes, and NK-cells were purified from 5 healthy subjects using Easy SepTM kit (#17961, #19044, #17995, STEMCELL). For cytofluorimetric analyses, PBMCs were labeled using CD45-KO, CD3-APC700, CD14-PC5.5, CD19-PC7, CD56-APC750, CD45–56–19–3 tetrachrome, and HLADR-PC5 antibodies (Beckman coulter). Kaluza software (Beckman Coulter) was used for the determination of the percentage of gated cells according to the gating strategy. *Cytokine evaluation:* For the cytokine expression evaluation, 200 ng/mL of LPS from *Salmonella*, *Fusobacterium nucleatum*, and OPS from *Fusobacterium nucleatum* were added to 5 × 10^5^ PBMC from 3 healthy donors (male, median age 32 years) for o/n incubation at 37 °C. Cells were harvested and cytokine expression levels were evaluated in the medium using a LEGENDplex Human Inflammation Panel (13-plex), according to the manufacturer instruction (740118, Biolegend), using Cytoflex (Beckman Coulter).

### Protein Expression and Purification

The full extracellular domain (FED) of Siglec-7 (Gly18-Gly354) containing a C-terminal histidine tag was expressed in suspension-adapted human HEK293S GnTI- cells, as previously published.^28^ The carbohydrate recognition domain (CRD) of Siglec-7 (Gly18-His148) containing a C-terminal histidine tag was expressed in LB and M9 culture medium. The protein resulted in the inclusion bodies, which were resuspended in 8 M urea lysis buffer; then, the soluble protein was subjected to IMAC purification using a HisTrap FF. The protein was refolded and then purified by a size-exclusion chromatography on a HiLoad 26/60 Superdex 75 pg (GE Healthcare) coupled on an AKTA Go FPLC system. Details related to expression and purification of Siglec-7 in both *E. coli* and HEK293 cells have been submitted elsewhere.^19^

### NMR Protein Assignment

HNCA, HNCACB, HNCO, and CBCAcoNH triple resonance experiments were recorded for the Siglec-7 CRD NMR assignment at 298 K on a Bruker’s Avance NEO 900 MHz spectrometer, equipped with a TCI cryo-probe. 3D HNcaCO was recorded at 298 K on a Bruker Avance 500 MHz spectrometer.^27^ Amino acid sequence from Y26 to T147 was assigned for the 93% excluding the 5 proline residues. Data acquisition and processing were performed on TOPSPIN 4.1.1 software and spectra were analyzed by using CARA (Computer Aided Resonance Assignment) software.^29^

### Protein-Based NMR Experiments

Protein-based NMR titration of Siglec-7 with OPS was performed by recording 2D ^1^H–^15^N HSQC NMR experiments on an aqueous buffered solution of 200 μM [U–^15^N] Siglec-7 CRD in 200 μL (20 mM potassium phosphate, pH 7.4, 50 mM NaCl, 0.01% NaN_3_, 1 mM of protease inhibitors, 10% D_2_O) in a 3 mm NMR tube. The spectra were recorded on the free protein and after the addition of increasing aliquots of OPS.

A stock solution of 3.2 mg of OPS from *F. nucleatum* ATCC 10953 dissolved in 800 μL of Milli-Q water was prepared and the solution was aliquoted in different fractions to reach 0.075, 0.075, 0.45, 0.6, 1.2, and 1.1 mg after lyophilization. Each of the lyophilized aliquots was subsequently redissolved in 200 μL of a sample of [U–^15^N] Siglec-7 CRD 200 μM (or the previous titration point) to reach final concentrations of ligand of 25, 50, 200, 400, 800, and 1160 μM assuming an average molecular weight of 15000 kDa for the OPS. Details are shown in Table S1. Experiments were acquired on a Bruker’s AVANCE NEO 900 MHz spectrometer equipped with a triple resonance TCI cryo-probe. The spectra were acquired using 32 scans, 2048 data points in the direct dimension, 128 data point in the indirect dimension, and recycle delay of 1.2 s and the temperature was kept at 298 K. Data acquisition and processing were performed with TOPSPIN 4.1.1 software and the spectra were analyzed using CARA.^29^ Chemical shift perturbations (CSP) were evaluated with the formula:^30^ $\Delta \delta =\frac{1}{2}\sqrt{{\Delta \delta }_{H^{2}}+{\left(\frac{{\Delta \delta }_{N}}{5}\right)}^{2}}$

### Ligand-Based NMR Experiments

All NMR experiments were recorded at 298 K on a Bruker AVANCE NEO 600-MHz spectrometer equipped with a cryo-probe. Data acquisition and processing were performed using TOPSPIN 4.1.1 software. Samples were prepared in phosphate-buffered saline (10 mM Na_2_HPO_4_, 2.7 mM KCl, 137 mM NaCl, 10 mM NaN_3_, pH 7.4) at 298 K, using D4 propionic acid sodium salt (TSP, 0.05 mM) as the internal reference. The NMR experiments were conducted with a protein ratio of 1:50. Saturation transfer difference (STD) NMR experiments: STD NMR spectra were acquired using 32k data points, zero-filled to 64k before processing. Protein resonances were selectively irradiated with 40 G pulses lasting 50 ms, with the off-resonance pulse frequency set at 40 ppm and the on-resonance pulses at 0 ppm. To suppress protein signals, a 20 ms spinlock pulse was applied. The acquisition parameters included 65k data points and 112 scans. The STD NMR experiments were performed at a saturation time of 2 s. Ligand epitope mapping was achieved by calculating the ratio (*I*_0_ – *I*_sat_)/*I*_0_, where *I*_sat_ corresponds to the intensity of the STD NMR signal and *I*_0_ to the intensity of the off-resonance spectrum. The strongest STD response was normalized to 100%, and all other proton STD signals were scaled accordingly. Control experiments were also performed in the absence of the protein to ensure the specificity of the observed STD signals. Transferred NOESY (tr-NOESY) experiments: Transferred NOESY spectra were recorded with data sets of 2048 × 512 pointsto analyze the interproton distances within the ligand, with mixing times ranging from 100 to 400 ms. Carr–Purcell–Meiboom–Gill (CPMG) NMR experiments were recorded to measure T_2_ spin–spin relaxations of the ligand in absence and in the presence of the protein. A pseudo-CPMG 2D sequence with water suppression using excitation sculpting with gradients was considered. A fixed echo time was set to 3 ms and a recycle delay to 2 s. T_2_ signals were analyzed with Dynamic Center 2.7.1 software by fitting the equation *f*(*t*) = *I*_o_ × exp (−*t*/*T*).

### Parametrization

The nonparametrized AAT unit was parametrized using a custom protocol with Gaussian 09,^31^ performing a restrained electrostatic potential (RESP) charge calculation with a Hartree–Fock method and a 6–31G* basis set. The .prep and .frcmod files were generated by combining Antechamber and xLeap.^32^ Trajectory analysis was conducted using the ptraj module in AMBER 18,^33^ and the molecular dynamics MD results were visualized with the VMD program.^33^

### Molecular Mechanics

Molecular mechanics calculations were performed using the MM3* force field available in the MacroModel 8.0 software from the Maestro package.^34^ A dielectric constant of 80 was applied to simulate vacuum conditions, and the disaccharide structures were explored by incrementally varying the Φ and Ψ angles with an 18° grid step. Each (Φ, Ψ) point was optimized using 2000 conjugate gradient steps.

### Molecular Dynamics Simulations

Molecular dynamics simulations were conducted with the AMBER 18 suite, employing specific force fields: ff14SB for the protein^35^ and Glycam06j-1 ^36^ for the saccharide portion of the ligands and TIP3P for the water molecules. A glycam-adapted force field was prepared for the AAT unit. Proteins were prepared using the Maestro Protein Preparation Wizard, which added missing hydrogens, adjusted the protonation states of ionizable groups, and capped the termini. Systems were hydrated with a truncated-octahedral box of TIP3P water, with a 15 Å buffer, and counterions were added to neutralize the system. Input files were generated using the tleap module of AMBER 18. Initial energy minimization was performed using the Sander module, while molecular dynamics simulations were carried out with the PMEMD module. Periodic boundary conditions and the particle mesh Ewald method were applied to represent electrostatic interactions, using a grid spacing of 1 Å. The initial minimization was conducted with the complex fixed followed by minimization of the entire system. Subsequently the system was gradually heated from 0 to 300 K, starting at constant volume and then transitioning to an isobaric ensemble. The temperature was maintained at 300 K for 50 ps with progressive energy minimizations and solute restraints. After the equilibration phase, restraints were removed, and the simulations proceeded in an isothermal–isobaric ensemble during the production phase. System coordinates were saved every 2 ps, generating a set of 10,000 structures per complex for further analysis.

Trajectories were analyzed using the ptraj module in AMBER 18, and the molecular dynamics results were visualized with the VMD program.^33^ Cluster analysis based on ligand RMSD was performed using the K-mean algorithm in the ptraj module. The representative structure of the most populated cluster was used to illustrate the complex interactions. Hydrogen bond determination was carried out with the CPPTRAJ module,^37^ defining a hydrogen bond as having a maximum distance of 3 Å between donor and acceptor atoms and a minimum A–H–D angle of 135°. 3D images were prepared using PyMOL,^38^ and dihedral conformation analysis was performed using a custom script that generated histograms of the most populated values during the simulation.

## Supplementary Material



## Notes

Supporting figures (description of Siglec-7 CRD, NMR spectra of the oligomers, binding studies, STD NMR spectra on 3-Mer, computational studies) and tables (cellular and relaxation experiments) (PDF)
