# Supplementary material for: Fusobacterium nucleatum Lipopolysaccharides O‑Antigen Defines a Novel Siglec‑7 Binding Epitope
Source: JACS Au. 2025 Sep 25;5(11):5367–80. doi: 10.1021/jacsau.5c00810 (PMC12648312; doi:10.1021/jacsau.5c00810)
Supplement: Supplementary file 1 [file au5c00810_si_001.pdf]

Supporting Information

Fusobacterium nucleatum LPS O-antigen defines a novel Siglec-7 binding epitope

Cristina Di Carluccio[1,2]‡, Ferran Nieto-Fabregat[1]‡, Linda Cerofolini[3], Celeste Abreu[4], Luis Padilla-Cortés[3], Giulia Roxana Gheorghita[3,5], Alessandro Antonio Masi[1], Lorena Buono[6], Manasik Gumah Adam Ali[7], Dimitra Lamprinaki[7], Antonio Molinaro[1,2], Nathalie Juge[7], Giovanni Smaldone[6], Ondřej Vaněk[4], Marco Fragai[3,5], Roberta Marchetti[1]*, Alba Silipo[1,2]*

1 Department of Chemical Sciences, University of Naples Federico II, Via Cinthia 4, 80126, Naples, Italy; roberta.marchetti@unina.it; silipo@unina.it
2 CEINGE-Biotecnologie Avanzate Franco Salvatore, Via Gaetano Salvatore 486, 80145, Napoli, Italy
3 Magnetic Resonance Centre (CERM), CIRMMP and Department of Chemistry "Ugo Schiff", University of Florence, Via Luigi Sacconi 6, 50019, Sesto Fiorentino, Italy
4 Department of Biochemistry, Faculty of Science, Charles University, Hlavova 2030/8, 12800, Prague, Czech Republic
5 Giotto Biotech s.r.l., Sesto Fiorentino, 50019, Italy
6 IRCCS SYNLAB SDN, Via G. Ferraris 144, 80146 Naples
7 The Food, Microbes and Health Institute Strategic Programme, Norwich Research Park, Quadram Institute Bioscience, Norwich, UK

‡ These authors contributed equally to this work

Corresponding author E-mail: roberta.marchetti@unina.it (RM), silipo@unina.it (AS)

Table of Contents

Supporting Figures pag 2
Supporting Tables pag 14

Scheme S1. Structural features of human Siglecs. The extracellular domain is composed of the N-terminal V-set immunoglobulin (Ig) domain that represents the sialic acid–binding site, followed by 1–16 C2 Ig domains. Most Siglecs exhibit characteristic regulatory motifs in the cytoplasmatic portion, as indicated in the legend. All Siglecs contain a conserved arginine that establishes a key salt bridge with the carboxyl group of the sialic acid.

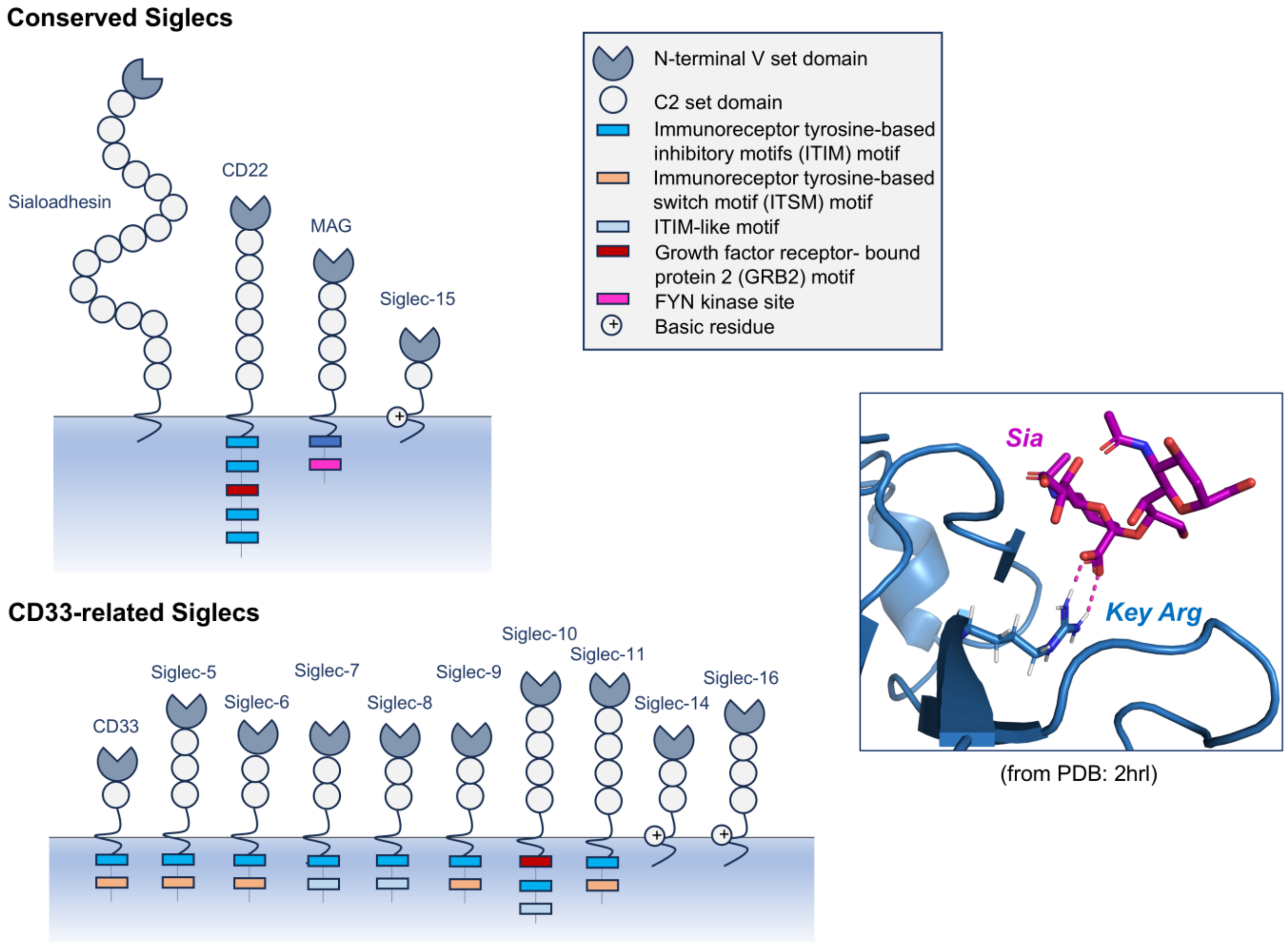

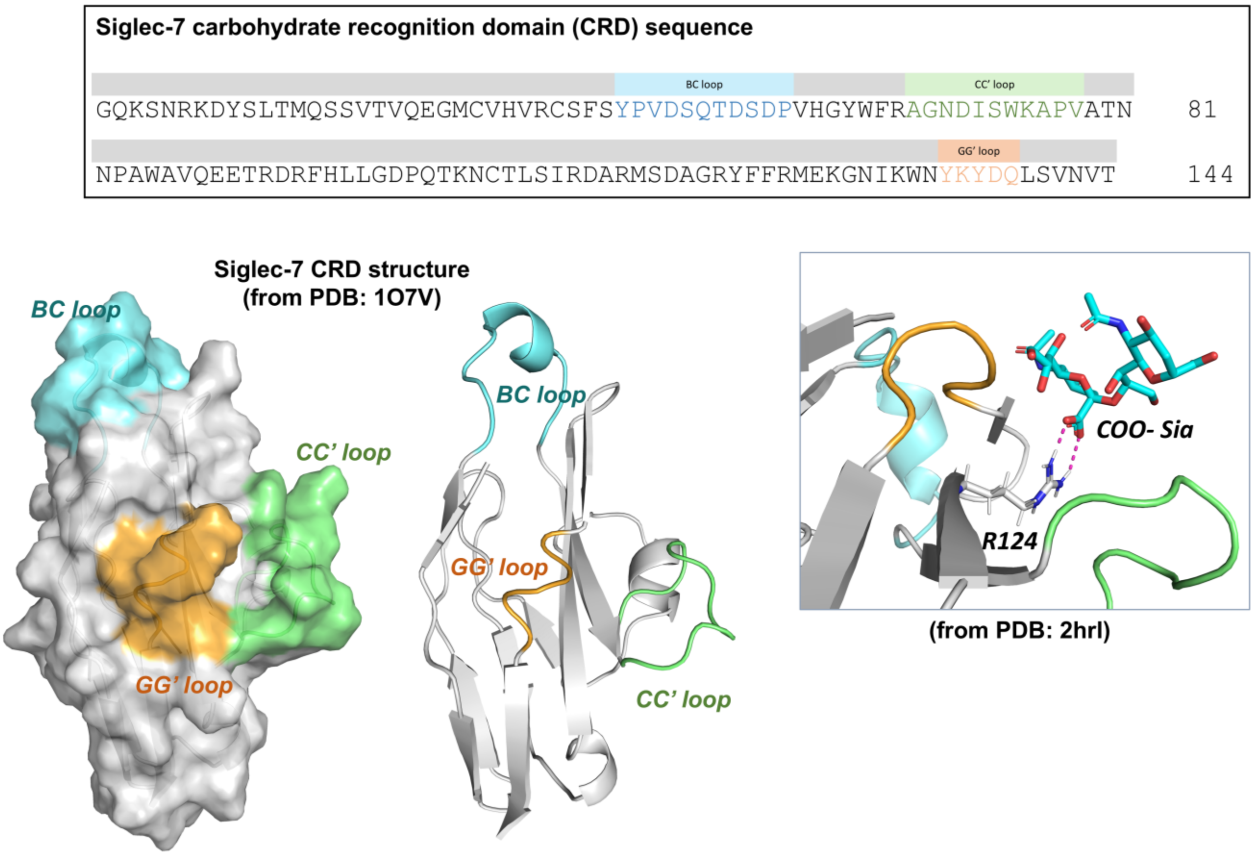

Figure S1. Siglec-7 carbohydrate recognition domain (CRD) sequence and 3D structure from PDB 1O7V. The BC, CC' and GG' loops are colored in cyan, green, and orange, respectively, and the key arginine R124 is evidenced in establishing the salt bridge with COO- of Sia (PDB: 2hrl).

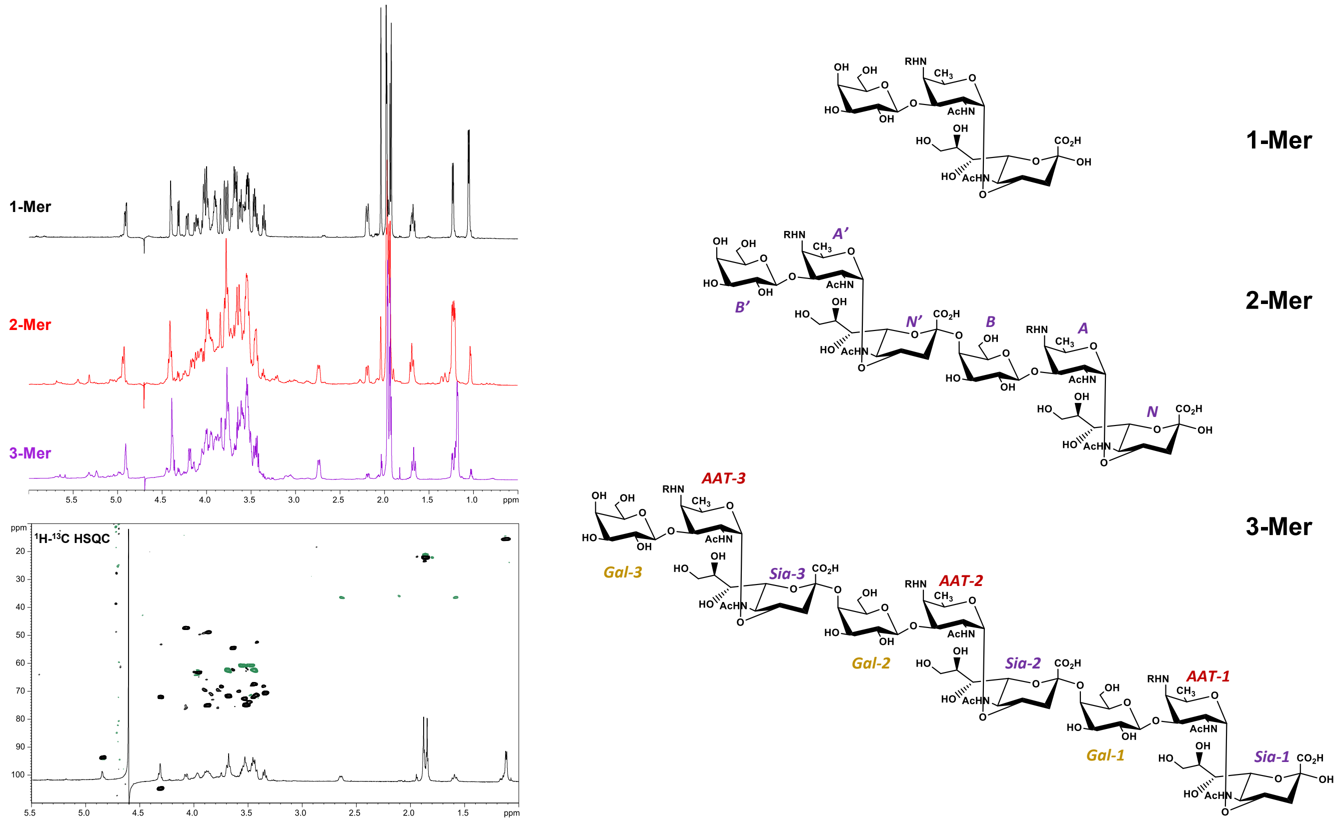

Figure S2. 1H NMR spectra and chemical structures of the oligomers - tri- (1-Mer), hexa- (2-Mer) and nonasaccharide (3-Mer) – obtained from mild acid hydrolysis of *Fn*10953 O-antigen which have been used for the interaction studies. The superimposition of 1D ^{1}H NMR and ^{1}H-^{13}C HSQC spectra of *F. nucleatum* 10953 polysaccharide has also been reported.

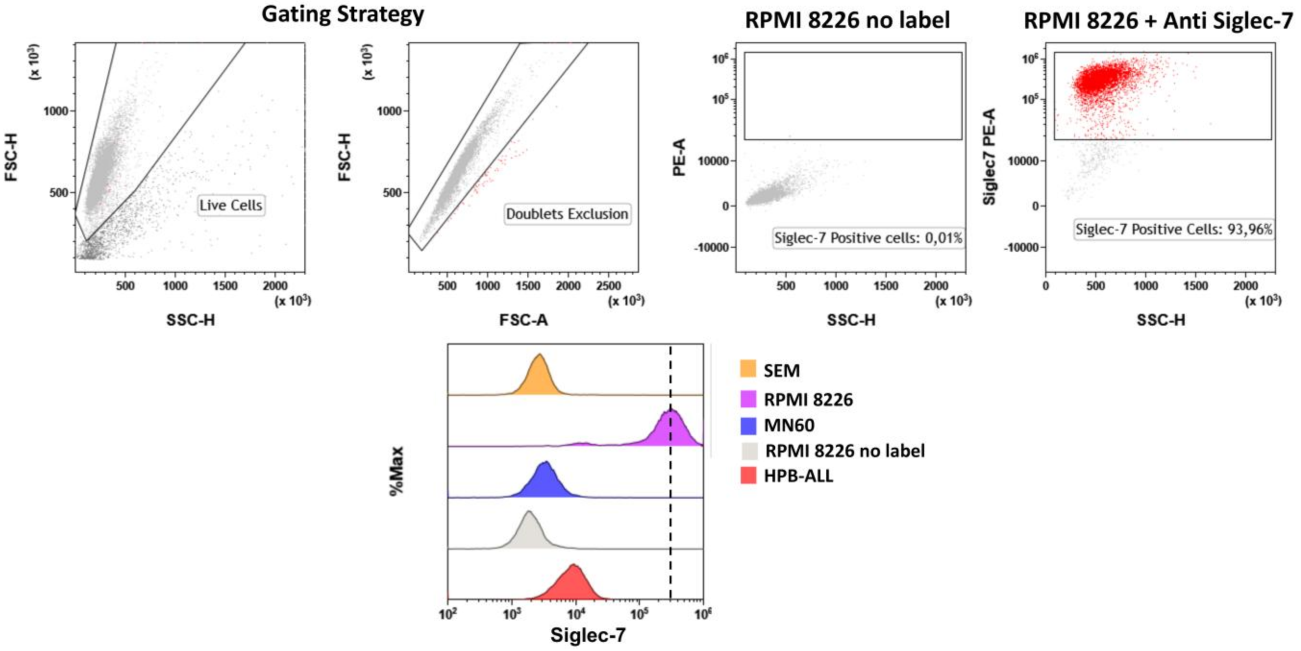

Figure S3. An exemplificative gating strategy of hematopoietic cell lines used to evaluate surface Siglec-7 protein. The lower panel reports the cytofluorimetric analyses of Siglec-7 protein levels, in RPMI 8226 (magenta), MN60 (blue), and HPB-ALL (ligh red), with respect to the unlabeled RPMI 8226 (grey).

A

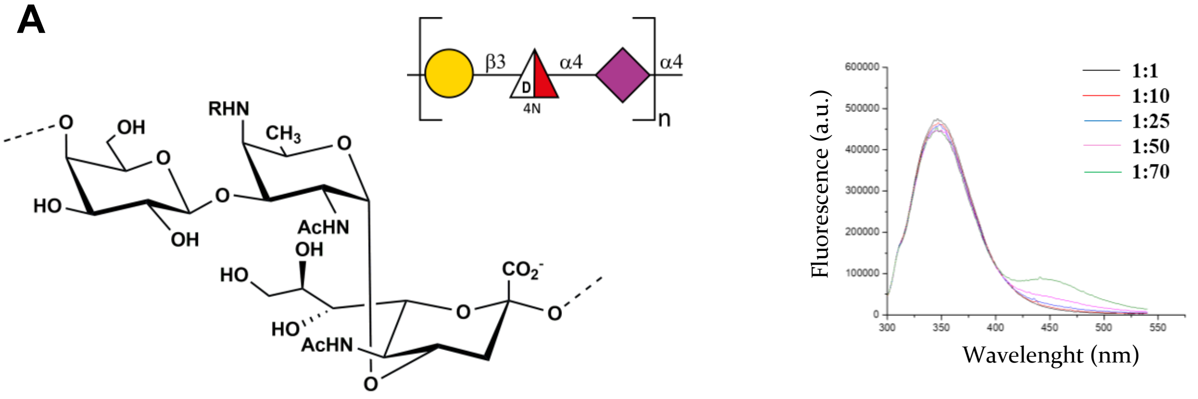

B

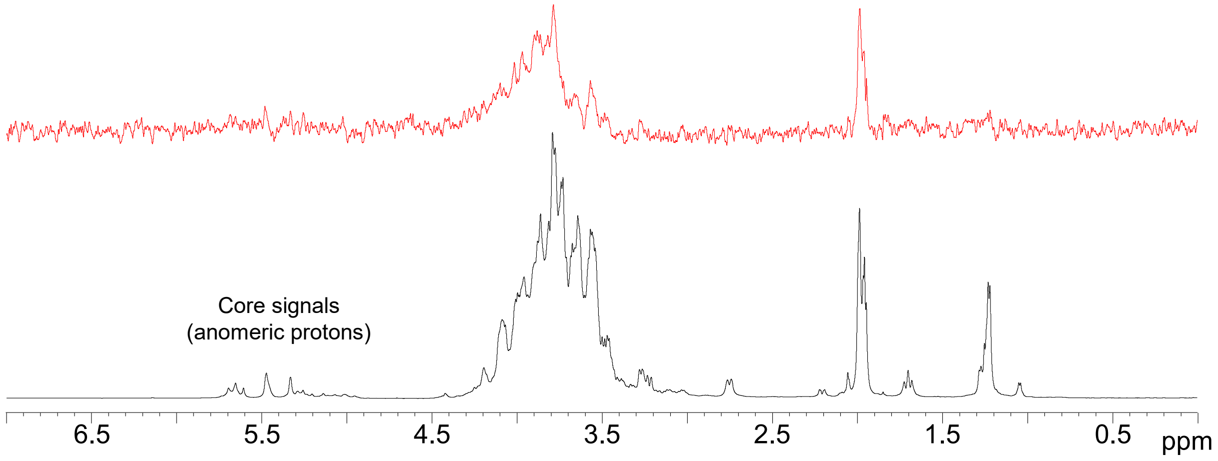

Figure S4. Fluorescence intensity of Siglec-7 upon the addition of increasing concentrations of 1-Mer. **A**) The absence of intensity variations indicated that 1-Mer was not recognized by Siglec-7. **B)** STD NMR spectrum of Siglec-7 with *Fn*10953 full polysaccharide STD NMR. The absence of STD signals coming from the protons of the core revealed the non-involvement of the core oligosaccharide moiety in the binding with Siglec-7.

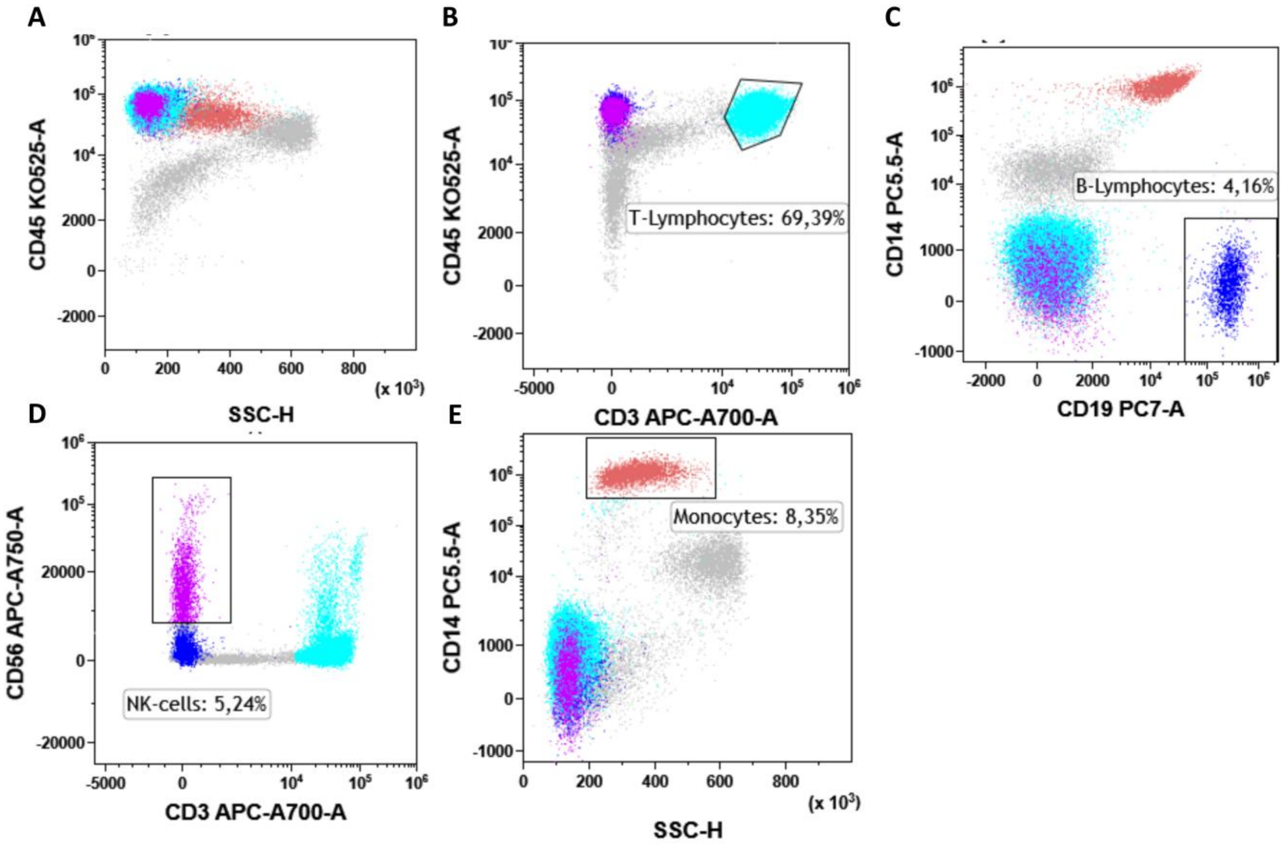

Figure S5. The gating strategy of cells reported in Figure 2A. From CD45+ cells (A), T-lymphocytes were gated as CD45+/CD3+ cells (B), B-lymphocytes were gated as CD19+/CD14- cells (C), NK cells as CD3-/CD56+ cells (D) and monocytes as CD14+ cells (E).

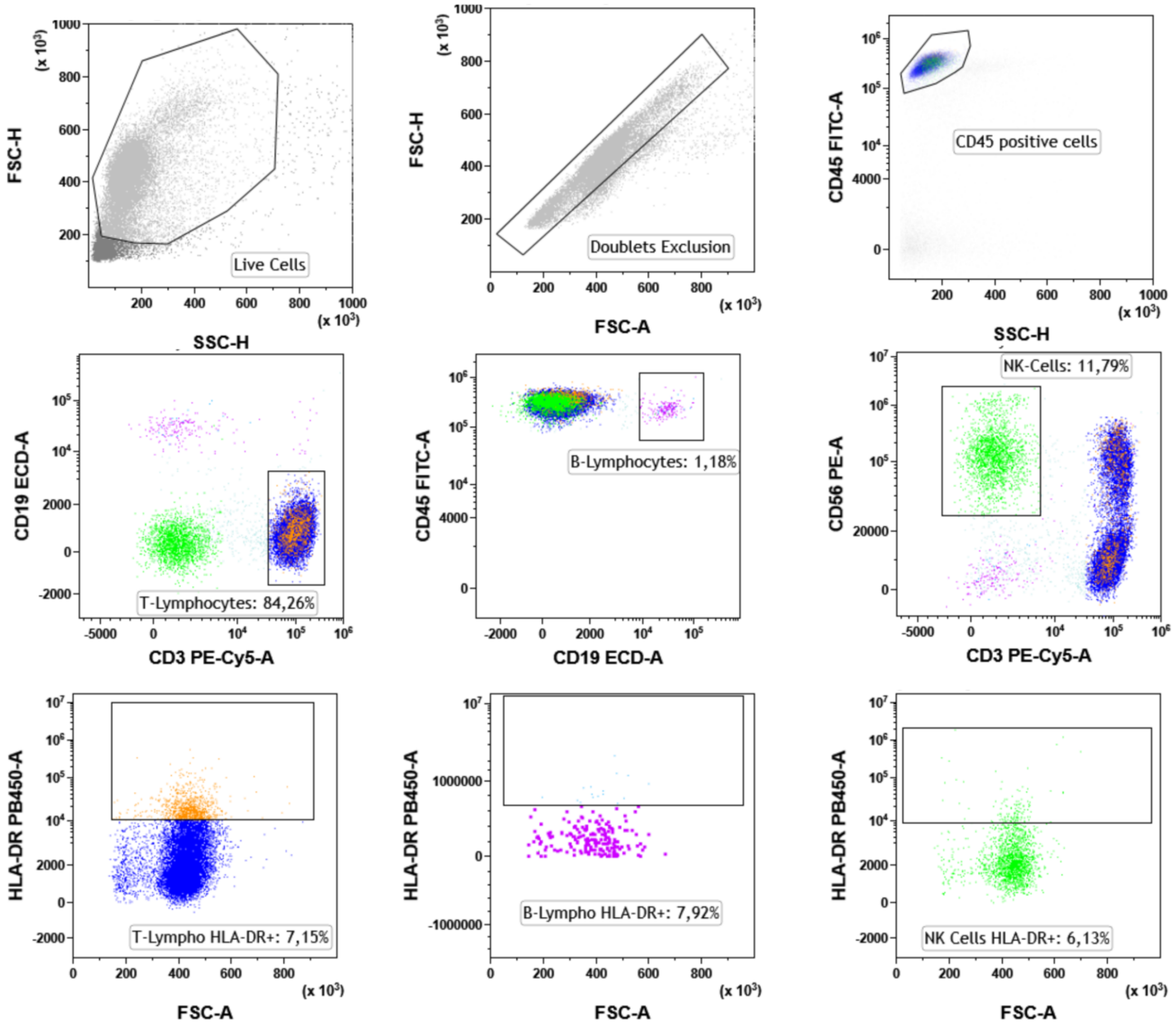

Figure S6. The gating strategy of cells reported in Figure 2B-E.

A

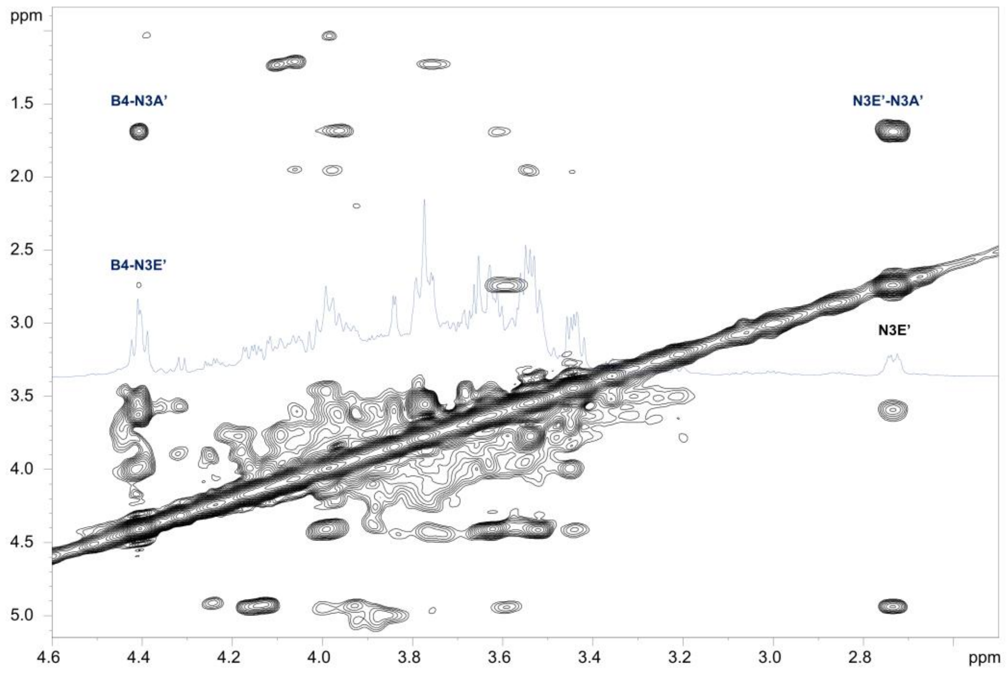

B

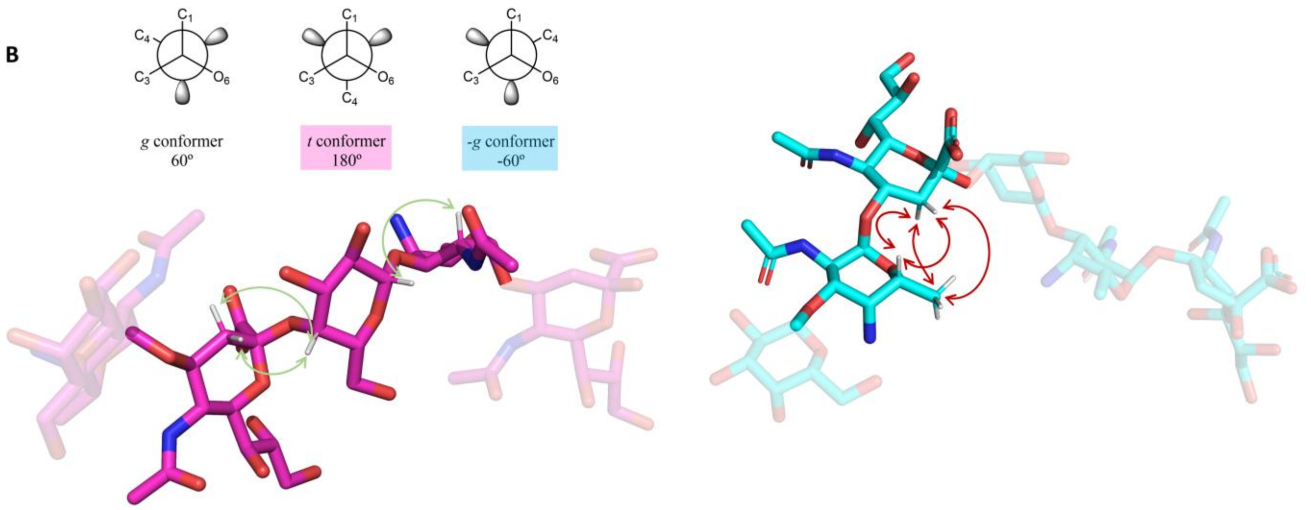

Figure S7. Free state conformational studies. **A)** 2D NOESY NMR spectrum of *Fn*10953 2-Mer in its free state. **B)** Newman projection of *g*, *t* and *-g* conformers. t conformer (in pink) with green arrows indicating the observed NOEs, whereas *-g* conformer (in cyan) with red arrows indicating the missing NOE that justifies *t* conformer.

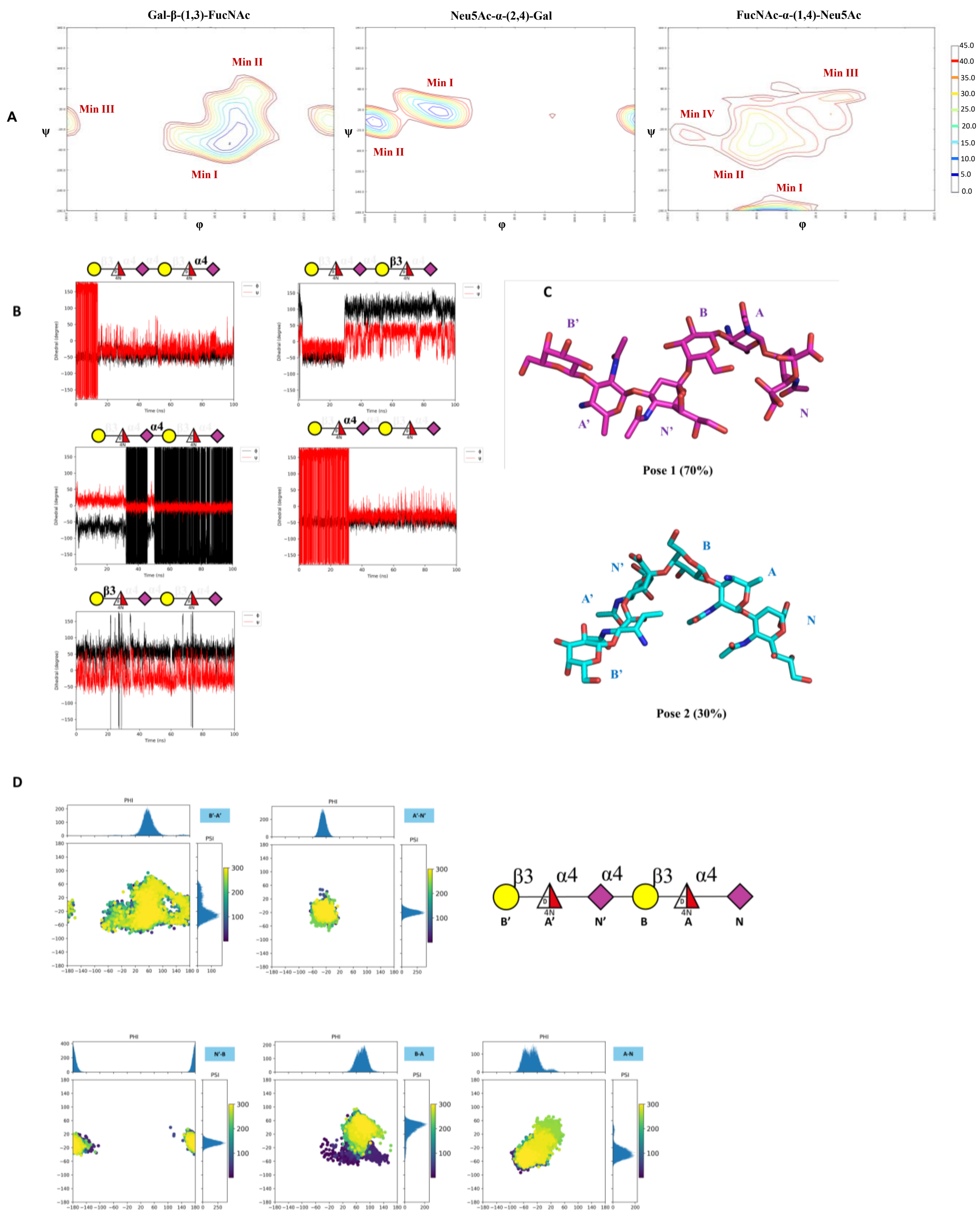

Figure S8. A) Adiabatic energy maps obtained by molecular mechanics simulations of the glycosidic torsion angles ϕ/ψ, of the basic constituent disaccharides. **B)** Torsion angles around the different glycosidic linkages monitored in a 300 ns MD simulation of the free state. **C)** Representation of the two observed families. The pose1 in cyan with a ϕ = 180º corresponding to the *t* conformer and the pose2 with a ϕ = 0º in magenta corresponding to the *g* conformer. **D)** 2-Mer bound state dihedral angles analysis represented as scatter plots of the φ torsion against ψ, for the 300 ns MD simulation with the relative histograms representing the most populated energies.

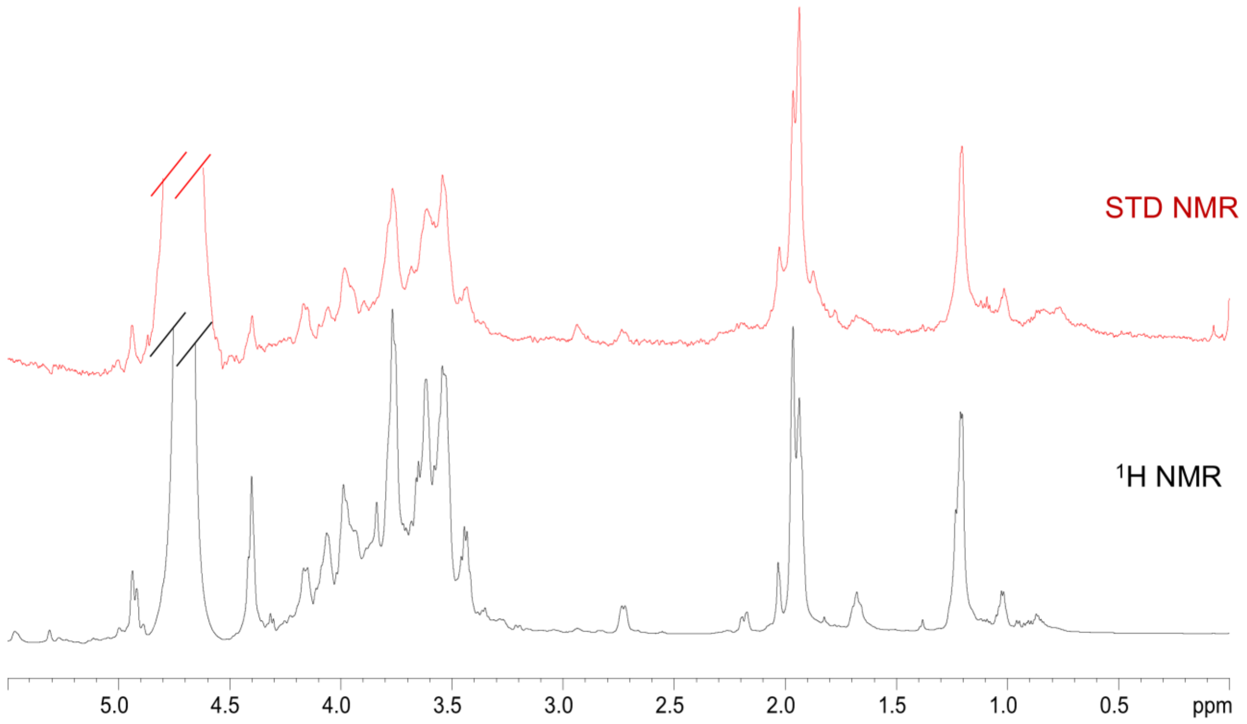

Figure S9. STD NMR spectrum of Siglec-7 and *Fn*10953 3-Mer.

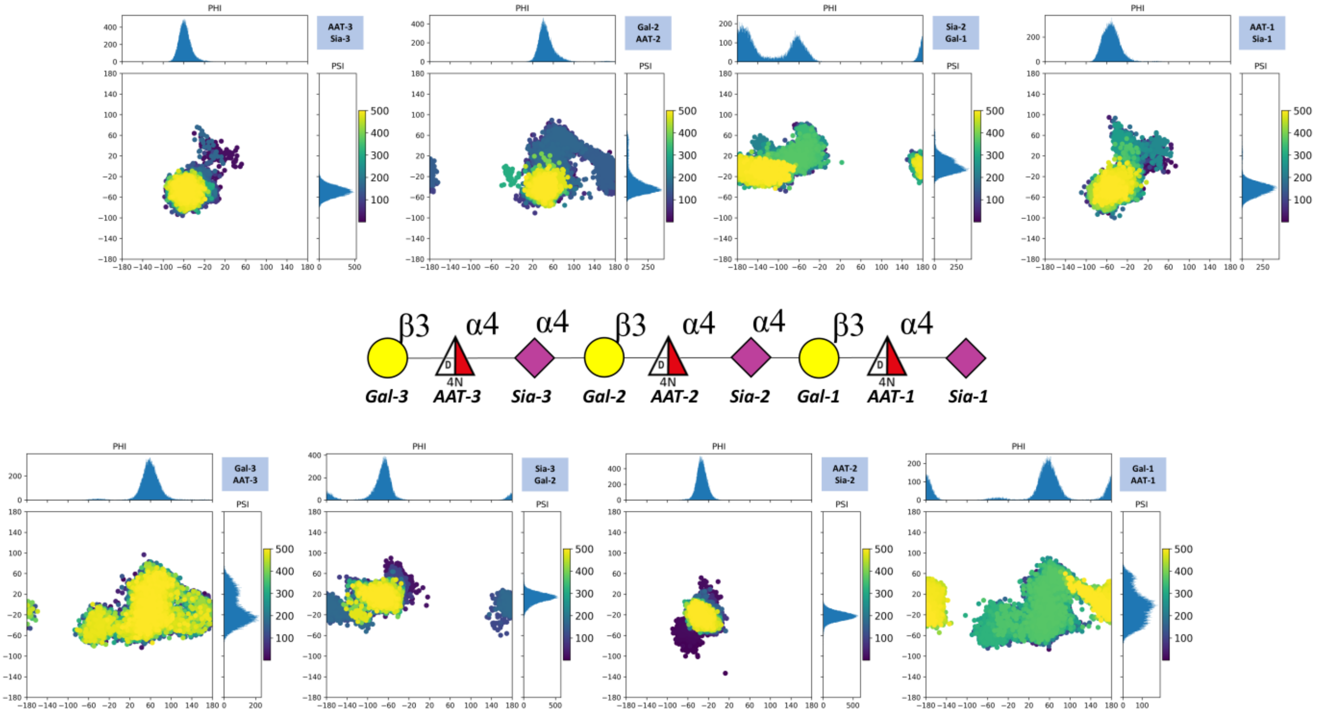

Figure S10. 3-Mer bound state dihedral angles analysis represented as scatter plots of the φ torsion against ψ, for the 500 ns MD simulation with the relative histograms representing the most populated energies.

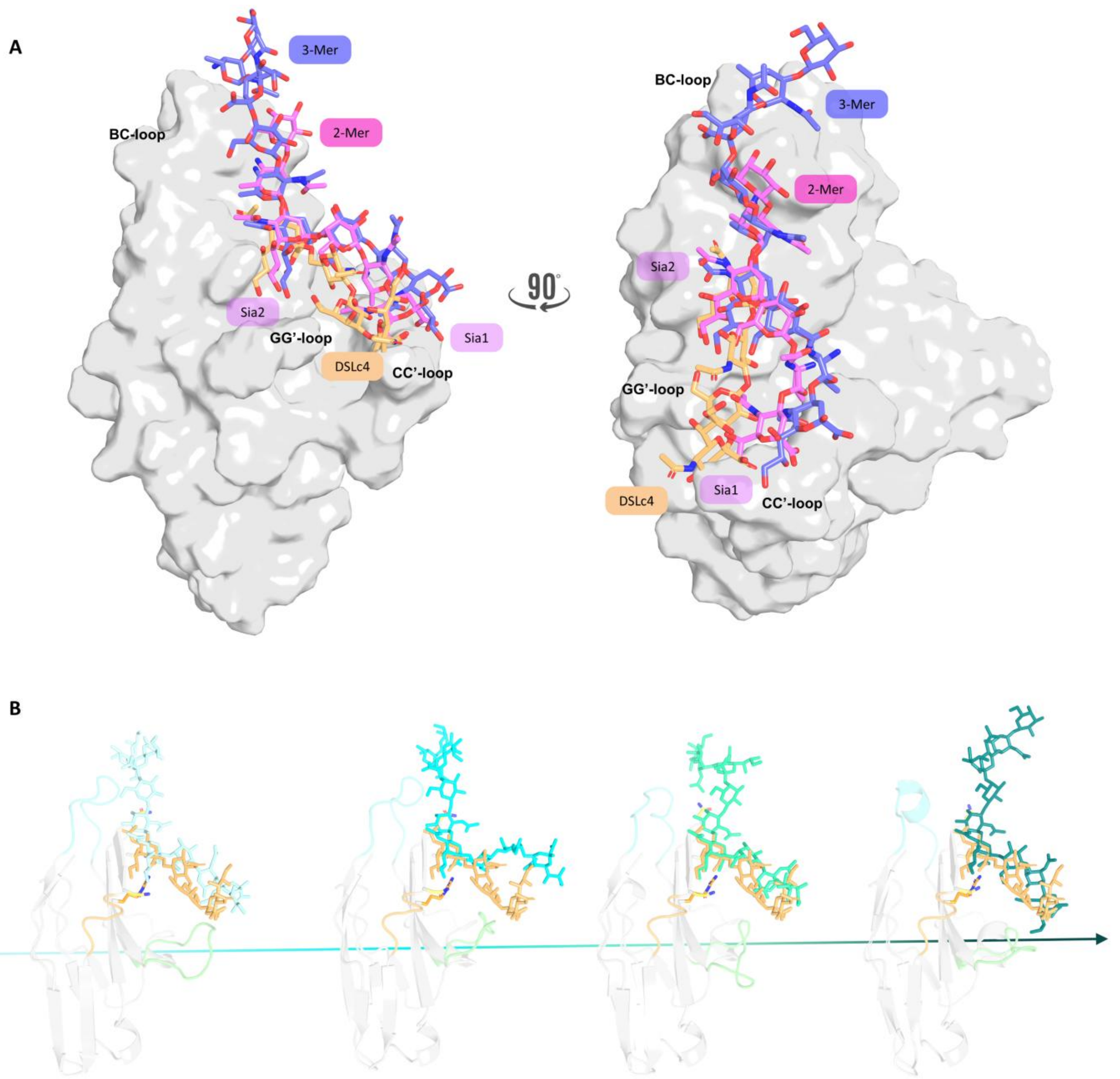

Figure S11. DSLc4 and *Fn*10953 LPS comparison in the interaction with Siglec-7. A) Structural representation of the interaction between the protein and the 3-Mer, 2-Mer, and DSLc4 displayed on the protein surface. The protein is shown as a grey surface with key loops involved in the interaction (BC-loop, GG'-loop, and CC'-loop) labelled. The ligand binding orientation is shown in two views rotated by 90°. Sia1 and Sia2 are labelled. The representation illustrates the spatial arrangement and molecular recognition of the ligands within the binding pocket. B) Comparison along the MD simulation with Siglec-7 between 3-Mer and DSLc4. It can be observed that the 3-Mer and DSLc4 not only interact differently with the CC'-loop but also that the 3-Mer preferentially interacts with the BC loop during its 'wing-like' movement.

Table S1. Two-way anova Tukey's multiple comparisons test

**T-Lympho**	**Mean Diff,**	**95% CI of diff,**	**Significant?**	
No Activation vs. LPS Salmonella	-1,352	-7,490 to 4,786	No	**ns**
No Activation vs. LPS Fuso 10953	-2,240	-8,378 to 3,898	No	**ns**
No Activation vs. OPS Fuso 10953 17/1	-1,218	-7,356 to 4,920	No	**ns**
LPS Salmonella vs. LPS Fuso 10953	-0,8880	-7,026 to 5,250	No	**ns**
LPS Salmonella vs. OPS Fuso 10953 17/1	0,1340	-6,004 to 6,272	No	**ns**
LPS Fuso 10953 vs. OPS Fuso 10953 17/1	1,022	-5,116 to 7,160	No	**ns**
**B-Lympho**	**Mean Diff,**	**95% CI of diff,**	**Significant?**	
No Activation vs. LPS Salmonella	3,002	-3,136 to 9,140	No	**ns**
No Activation vs. LPS Fuso 10953	-1,902	-8,040 to 4,236	No	**ns**
No Activation vs. OPS Fuso 10953 17/1	2,402	-3,736 to 8,540	No	**ns**
LPS Salmonella vs. LPS Fuso 10953	-4,904	-11,04 to 1,234	No	**ns**
LPS Salmonella vs. OPS Fuso 10953 17/1	-0,6000	-6,738 to 5,538	No	**ns**
LPS Fuso 10953 vs. OPS Fuso 10953 17/1	4,304	-1,834 to 10,44	No	**ns**
**NK-Cells**	**Mean Diff,**	**95% CI of diff,**	**Significant?**	
No Activation vs. LPS Salmonella	-3,972	-10,11 to 2,166	No	**ns**
No Activation vs. LPS Fuso 10953	-3,776	-9,914 to 2,362	No	**ns**
No Activation vs. OPS Fuso 10953 17/1	-6,748	-12,89 to -0,6095	Yes	*
LPS Salmonella vs. LPS Fuso 10953	0,1960	-5,942 to 6,334	No	**ns**
LPS Salmonella vs. OPS Fuso 10953 17/1	-2,776	-8,914 to 3,362	No	**ns**
LPS Fuso 10953 vs. OPS Fuso 10953 17/1	-2,972	-9,110 to 3,166	No	**ns**

Table S2. Relaxation Experiments. T1 and T2 values of 3-Mer in the free (*f*) and bound (*b*) states.

F2 [ppm]	T_1f [s]	$Error_f$	T_1b [s]	$Error_b$	Fit Info
4.933	1.60	0.13	1.52	0.13	Done
4.411	1.67	0.08	1.37	0.06	Done
4.150	2.00	0.10	1.75	0.11	Done
4.063	1.22	0.05	1.08	0.04	Done
3.843	1.36	0.07	1.33	0.04	Done
2.024	1.74	0.07	1.45	0.06	Done
1.963	2.14	0.05	1.86	0.04	Done
1.934	2.31	0.08	2.05	0.09	Done
1.676	0.87	0.09	0.76	0.07	Done
1.208	0.61	0.08	0.61	0.08	Done
1.020	0.41	0.04	0.39	0.05	Done

F2 [ppm]	T_2f [s]	$Error_f$	T_2b [s]	$Error_b$	Fit Info
4.939	0.10	0.03	0.09	0.02	Done
4.405	0.10	0.02	0.09	0.02	Done
4.158	0.12	0.02	0.12	0.01	Done
4.063	0.14	0.02	0.12	0.02	Done
3.843	0.14	0.02	0.12	0.01	Done
2.024	0.24	0.04	0.16	0.03	Done
1.963	0.20	0.03	0.19	0.02	Done
1.934	0.25	0.02	0.14	0.02	Done
1.676	0.11	0.01	0.09	0.01	Done
1.208	0.11	0.01	0.11	0.012	Done
1.020	0.12	0.03	0.16	0.013	Done
